# Design and Synthesis of Novel Thiazolo[5,4-d]pyrimidine Derivatives with High Affinity for Both the Adenosine A_1_ and A_2A_ Receptors, and Efficacy in Animal Models of Depression

**DOI:** 10.3390/ph14070657

**Published:** 2021-07-09

**Authors:** Flavia Varano, Daniela Catarzi, Erica Vigiani, Diego Dal Ben, Michela Buccioni, Gabriella Marucci, Lorenzo Di Cesare Mannelli, Elena Lucarini, Carla Ghelardini, Rosaria Volpini, Vittoria Colotta

**Affiliations:** 1 Dipartimento di Neuroscienze, Psicologia, Area del Farmaco e Salute del Bambino, Sezione di Farmaceutica e Nutraceutica, Universita’degli Studi di Firenze, Via Ugo Schiff 6, 50019 Sesto Fiorentino, Italy; daniela.catarzi@unifi.it (D.C.); erica.vigiani@unifi.it (E.V.); vittoria.colotta@unifi.it (V.C.); 2 Scuola di Scienze del Farmaco e dei Prodotti della Salute, Università degli Studi di Camerino, Via S. Agostino 1, 62032 Camerino, Italy; diego.dalben@unicam.it (D.D.B.); michela.buccioni@unicam.it (M.B.); gabriella.marucci@unicam.it (G.M.); rosaria.volpini@unicam.it (R.V.); 3 Dipartimento di Neuroscienze, Psicologia, Area del Farmaco e Salute del Bambino, Sezione di Farmacologia, Università degli Studi di Firenze, Viale Pieraccini 5, 50139 Firenze, Italy; lorenzo.mannelli@unifi.it (L.D.C.M.); elena.lucarini@unifi.it (E.L.); carla.ghelardini@unifi.it (C.G.)

**Keywords:** G protein-coupled receptors, adenosine A_1_ receptor ligands, adenosine A_2A_ receptor ligands, thiazolo[5,4-d]pyrimidines, ligand-adenosine receptor modeling studies, depression

## Abstract

New compounds with a 7-amino-2-arylmethyl-thiazolo[5,4-d]pyrimidine structure were synthesized and evaluated in vitro for their affinity and/or potency at the human (h) A_1_, hA_2A_, hA_2B_, and hA_3_ adenosine receptors (ARs). Several compounds (**5**, **8**–**10**, **13**, **18**, **19**) were characterized by nanomolar and subnanomolar binding affinities for the hA_1_ and the hA_2A_ AR, respectively. Results of molecular docking studies supported the in vitro results. The 2-(2-fluorobenzyl)-5-(furan-2yl)-thiazolo[5,4-d]pyrimidin-7-amine derivative **18** (hA_1_ K_i_ = 1.9 nM; hA_2A_ K_i_ = 0.06 nM) was evaluated for its antidepressant-like activity in in vivo studies, the forced swimming test (FST), the tail suspension test (TST), and the sucrose preference test (SPT) in mice, showing an effect comparable to that of the reference amitriptyline.

## 1. Introduction

Depression is a common debilitating illness ranked in many countries as the most prevalent of all psychiatric diseases [1]. According to the Diagnostic and Statistical Manual of Mental Disorders, depression is characterized by depressed mood and anhedonia (decreased interest in, and ability to experience, pleasure) associated with symptoms that may include significant weight gain or loss, insomnia, psychomotor agitation or retardation, fatigue or loss of energy, diminished ability to think or concentrate, and recurrent thoughts of death or suicide [2]. Moreover, depression may represent a risk factor for cancer [3], diabetes [4], and cardiovascular disorders [5,6], and shows comorbidity with several medical illness including serious CNS disorders such as Alzheimer’s (AD) and Parkinson’s disease (PD) [6,7], stroke [8], and chronic pain [9]. Over the years, different classes of antidepressants have been developed. However, the current treatments of depressed patients are still unsatisfactory. Particularly worrying is the fact that many patients only partially respond and some remain refractory. Moreover, commonly used medications are associated with adverse reactions difficult to tolerate including cardiovascular, anticholinergic, neurologic, gastrointestinal, and other side effects. Another big drawback of antidepressant therapy currently in use is that its full efficacy begins to appear only after 4–6 weeks of treatment or even more [10,11]. Thus, the search for novel therapeutic strategies for the treatment of depression disorders represents an important research priority.

Particular interest has been devoted to the adenosine signaling system as a new target for the treatment of depression [12,13]. Adenosine is an endogenous nucleoside present in all mammal tissues where it regulates several important functions. Adenosine exerts its actions by binding to different receptor subtypes, namely A_1_, A_2A_, A_2B_, and A_3_, all belonging to the G-protein coupled receptor family (GPCR). Upon stimulation, A_1_ and A_3_ adenosine receptors (ARs) inhibit adenylate cyclase activity, thus decreasing cAMP levels. Instead, A_2A_ and A_2B_ ARs stimulation increases adenylate cyclase activity and cAMP production [14]. In the central nervous system (CNS), adenosine plays an important role in the regulation of synaptic transmission and neuronal excitability. In the brain, several AR subtypes are present, with A_1_ and A_2A_ ARs being the most abundant. In the CNS, A_2A_ ARs are localized on neurons and glial cells and are expressed in almost all districts with the highest level found in the striatum. Adenosine A_1_ receptors are widely distributed in the brain with a high concentration, especially in the hippocampus. All these regions regulate processes such as cognition, motivation, and emotion, which appear to be altered in depressed patients [12,13].

Caffeine, which is a naturally occurring methylxanthine, is one of the most frequently used psychoactive substances. Caffeine is a weak non-selective A_1_ and A_2A_ AR antagonist, and has been demonstrated to influence behavior in classical animal models of depression [15]. In fact, caffeine after acute or repeated administration of a broad range of doses, increased mobility in the forced swim test (FST) and tail suspension test (TST). These screening tests are usually used to evaluate antidepressant drugs where the duration of immobilization is considered a measure of the depressive state. In accordance with the effects of caffeine, A_1_ and A_2A_ AR selective antagonists also provide antidepressant-like behavior. Thus, the single administration in mice of DPCPX, a selective A_1_ AR antagonist as well as of the well-known A_2A_ AR selective antagonists SCH 58261, ZM 241385, or istradefylline, exhibited antidepressant activity in the FST and TST [16,17,18]. Finally, it has been demonstrated that both caffeine and selective A_1_ or A_2A_ AR antagonists can enhance the antidepressant like activity of common antidepressant [16,19,20].

To conclude, it is important to mention the role of ARs in PD. In fact, much experimental data have been produced that demonstrate the efficacy of A_2A_ AR antagonists in different animal models of PD [21]. Moreover, the interest in the use of A_2A_ AR antagonists in PD has increased also due to their effectiveness in the treatment of non-motor symptoms of PD such as depression [22,23]. Preclinical animal models as well as clinical studies also demonstrated that non selective A_1_ and A_2A_ AR antagonists improved motor dysfunctions of PD, were neuroprotective through A_2A_ AR antagonism, and may also have enhanced cognitive functions via A_1_ AR antagonism [21,24,25].

**Figure 1:**
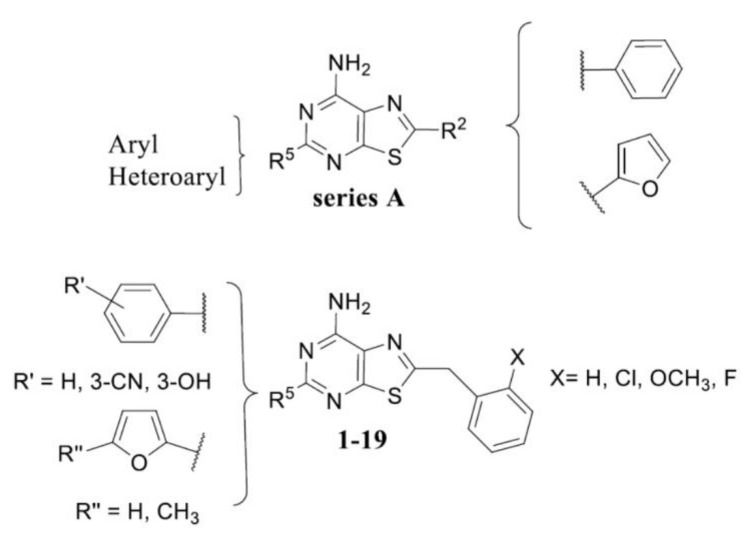
Previously (**series A**) and currently (**1**–**19**) reported 7-amino-thiazolo[5,4-d]pyrimidine derivatives.

Our efforts in the pursuit of novel AR antagonists have centered on mono- or bicyclic heteroaromatic systems. [26,27,28,29,30]. Within this research, an interesting class of antagonists/inverse agonists (i.e., the thiazolo[5,4-d]pyrimidine series) was identified [31,32,33,34,35]. Recently, we investigated the 7-amino-thiazolo[5,4-d]pyrimidine **series A** (Figure 1) featured by a phenyl or a furan-2yl ring at position 2 (R^2^) combined with an aryl or heteroaryl group at position 5 (R^5^) [34].

The results showed that compounds of **series A** are in general A_1_/A_2A_ AR dual antagonists and that their affinity can be modulated first by the nature of the substituent attached at position-2, and second, by that of substituents at position-5. Based on these findings, we herein report on the design and synthesis of new 7-aminothiazolopyrimidines **1**–**19** to obtain more potent A_1_ and/or A_2A_ AR antagonists. To this goal, compounds **1**–**19** were decorated at position-5 with selected substituents that in previously reported compounds of series A enhanced affinity toward A_1_ and/or A_2A_ AR (i.e., an unsubstituted or 3-CN or 3-OH substituted phenyl ring, and a heteroaryl moiety (furan-2yl or 5-methyl-furan-2yl) [34]), while at position-2, a benzyl or an ortho-substituted benzyl moiety was introduced since it improved A_2A_ AR affinity in other classes of our bicyclic dual A_1_/A_2A_ antagonists [26].

## 2. Results and Discussion

### 2.1. Chemistry

**Scheme 1:**
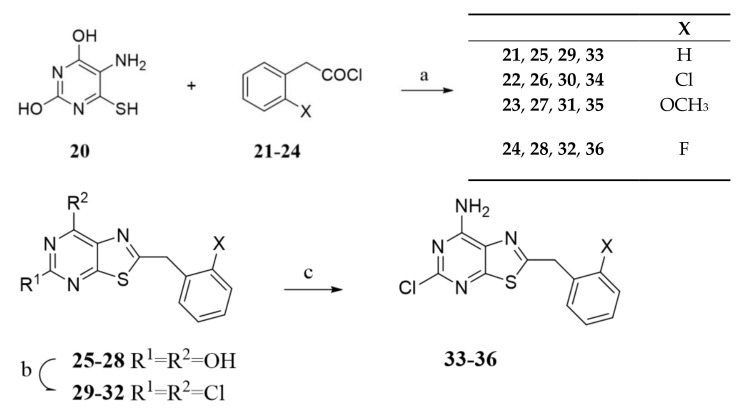
Reagents and conditions. (a) NMP, 150 °C, 10–15 h; (b) POCl_3_, 160 °C MW, 30 min; (c) NH_4_OH/EtOH, reflux, 6 h.

**Scheme 2:**
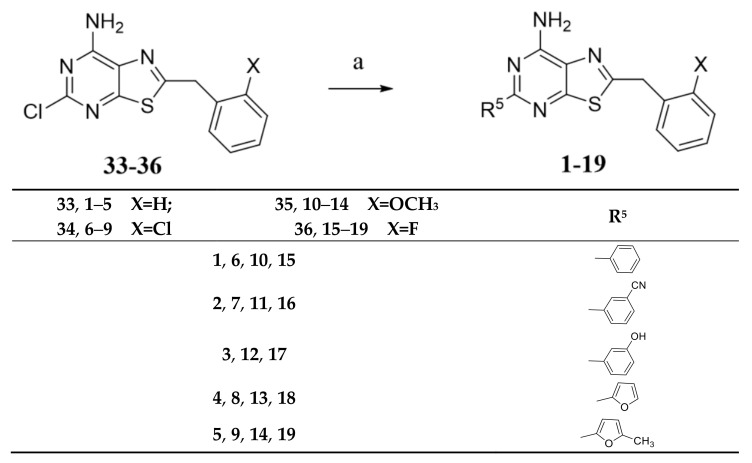
Reagents and conditions. (a) R^5^B(OH)_2_, tetrakis, Na_2_CO_3_, DME/H_2_O, reflux 4 h (**1**–**5**) or microwave irradiation, 160 °C, 30 min (**6**–**19**).

The synthesis of the reported molecules **1**–**19** was achieved as depicted in Scheme 1 and Scheme 2.

The bicyclic thiazolopyrimidine core suitably decorated at position-2 was first synthesized, then introduction of the diverse substituents at position-5 was performed. Briefly, by reacting 2-aminothiole **20** [36] with the suitable arylacetylchloride **21**–**24** [37,38,39] at high temperature, the corresponding thiazolo[5,4-d]pyrimidine-5,7-dihydroxy derivatives **25**–**28** were obtained (Scheme 1). The latter were chlorinated by reaction with POCl_3_ under microwave irradiation to the corresponding 5,7-dichloro bicyclic derivatives **29**–**32**, which in the presence of aqueous ammonia solution (33%) furnished the 7-amino-5-chloro substituted intermediates **33**–**36**. Finally, replacement of the 5-chloro group of **33**–**36** with the opportune R^5^ substituents was achieved by reacting compounds **33**–**36** and the suitable boronic acids under Suzuky conditions (Scheme 2).

### 2.2. Binding and cAMP Assays

Compounds **1**–**19** were evaluated for their binding affinity at hA_1_, hA_2A_, and hA_3_ ARs, stably transfected in Chinese hamster ovary (CHO) cells. Moreover, they were also tested at the hA_2B_ AR subtype by determining their inhibitory effects on 5′-(*N*-ethylcarboxamido) adenosine (NECA)-stimulated cAMP levels in hA_2B_ CHO cells (Table 1).

The results showed that we reached our goal, since all final compounds **1**–**19** featured by the presence of an unsubstituted or ortho-substituted benzyl ring at position 2 of the bicyclic core, exhibited good to high binding affinities at both the A_1_ and A_2A_ ARs higher than those of the previously reported compound **20** bearing a phenyl ring at the same position. Furthermore, compounds **1**–**19** were more selective toward these two receptor subtypes. In fact, affinity values at the A_3_ AR were comparable to or higher than that of reference **20**. Only three compounds (i.e., **5**, **9**, **18**) possessed a higher affinity and among them, compound **9** showed a K_i_ value in the nanomolar range (K_i_ = 8.5 nM). Finally, all the examined compounds blocked the A_2B_ AR with very low potencies spanning from IC_50_ = 384 nM to IC_50_ > 30,000 nM.

Analyzing the results in detail, eight compounds (**5**, **6**, **8**, **10**, **13**, **15**, **18**, **19**) showed single-digit nanomolar hA_1_ AR K_i_ values (1.9 nM < K_i_ < 6.8 nM), while two (compound **9** and **17**) fell in the subnanomolar range (K_i_ = 0.5 nM for both). All the other derivatives (**1**–**4**, **7**, **11**, **12**, **14**, **16**) possessed good to high hA_1_ AR binding affinity spanning from a K_i_ value of 10.2 nM to one of 72.8 nM.

Regarding the affinity at the A_2A_ AR, all the examined compounds were more active at this subtype than at the A_1_ AR. Moreover, compounds **1**–**19** exhibited hA_2A_ AR K_i_ values below 13.8 nM, and for eight of them (**5**, **6**, **8**, **9**, **10**, **13**, **18**, **19**), the K_i_ values fell in the subnanomolar range (0.06 nM < K_i_ < 0.8 nM). In conclusion, we identified several compounds (**5**, **6**, **8**, **9**, **10**, **13**, **18**, **19**) characterized simultaneously by nanomolar and subnanomolar binding affinities for the A_1_ and A_2A_ ARs, respectively. Among them, compound **18** emerged, exhibiting the highest hA_1_ and A_2A_ AR affinities (A_1_ K_i_ = 1.9 nM; A_2A_ K_i_ = 0.06 nM) combined with the highest selectivity toward these two receptor subtypes.

In general, better results in terms of binding affinities at both the A_1_ and A_2A_ ARs were found in the ortho-substituted 2-benzyl derivative groups (**6**–**9**, **10**–**14**, **15**–**19**) if compared to the corresponding unsubstituted 2-benzyl derivative group (**1**–**5**). However, it is difficult to establish which substituent (Cl, OCH_3_, F) on the 2-benzyl ring leads to more advantageous effects. Moreover, it seems that the presence of an unsubstituted phenyl as well as a furan-2yl or a 5-methyl-furan-2yl ring at position 5 of the bicyclic core, leads to compounds (**1**, **4**, **5**, **6**, **8**, **9**, **10**, **13**, **14**, **15**, **18**, **19**), showing high affinity for both the A_1_ and A_2A_ ARs regardless of the substituent at position 2.

Derivatives **5**, **8**–**10**, **13**, **18** and **19**, due to their high hA_2A_ AR affinity, were also tested to assess their ability to block the hA_2A_ AR by evaluating their effect on NECA induced increase of cAMP accumulation in CHO cells, stably expressing hA_2A_ ARs (Table 2). Compound **18** was also tested to evaluate its antagonist behavior at the hA_1_ AR by assessing its ability to counteract NECA-induced decrease of cAMP accumulation.

The data indicated that the tested compounds were potent A_2A_ AR antagonists showing IC_50_ values below 100 nM. In particular, **9** and **18**, in accordance with their binding data, resulted in the most potent compounds possessing a potency of the low nanomolar order (IC_50_ = 7.7 and IC_50_ = 14, respectively). IC_50_ value at hA_1_ AR of compound **18** indicated that it behaves as a quite potent hA_1_ AR antagonist (IC_50_ = 360 ± 115 nM).

### 2.3. Molecular Docking Studies

The binding mode at the hA_2A_ AR cavity of the newly synthesized compounds was simulated with the aid of computational tools. As a biomolecular target of docking analyses, the crystal structure of the hA_2A_ AR in complex with the antagonist/inverse agonist ZM241385 (pdb code: 5NM4; 1.7-Å resolution [40]) was chosen. MOE (Molecular Operating Environment August 2016 [41]) software and the CCDC Gold [42] and Cresset Flare [43] docking tools were used for docking analyses. Analogue protocol was also employed to carry out docking studies at the hA_1_ AR. For this task, the crystal structure of the receptor in complex with the antagonist PSB36 (pdb code: 5N2S; 3.3-Å resolution) was selected [44].

**Figure 2:**
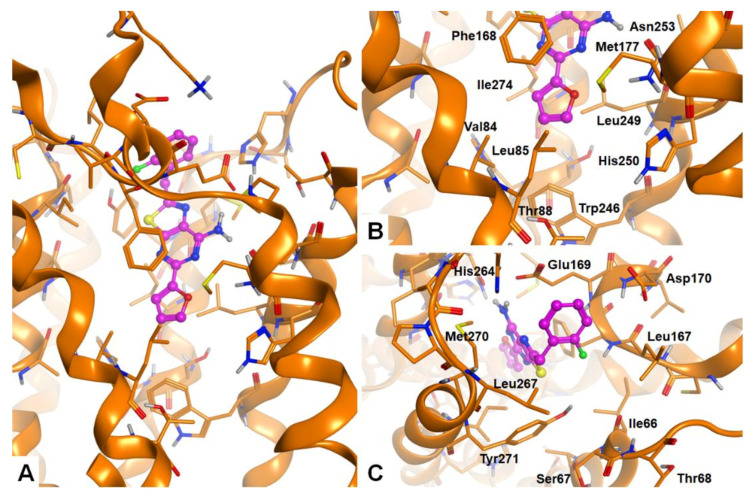
Docking-based hypothetical binding mode of the synthesized compounds at the hA_2A_ AR cavity; compound **18** is represented. (**A**) global view of the compound orientation within the receptor cavity; (**B**,**C**) detailed view of the 5- and 2-substituents, respectively, and the receptor residues located in their proximity.

As previously made for analogues of these compounds, docking analyses of the newly synthesized molecules were performed with various docking tools to obtain a sort of average binding mode prediction at the receptor binding site. The three docking tools provided analogous results. Docking conformation of compound **18** at the hA_2A_ AR cavity is reported in Figure 2 (see text for details).

Docking conformations make the new compounds being inserted in the binding cavity with the thiazolo[5,4-d]pyrimidine scaffold located between the side chains of Phe168 (EL2) and Leu249^6.51^, providing π–π interaction with these two amino acids (Figure 2). The exocyclic amine group makes polar interaction with Asn253^6.55^ and Glu169 (EL2) side chains, while the N6 atom makes an additional polar interaction again with Asn253^6.55^. The 2-substituent (an unsubstituted or substituted benzyl group) points toward the extracellular space and is located in proximity to Ile66^2.64^, Ser67^2.65^, Thr68^2.66^, Leu167 (EL2), Phe168 (EL2), Glu169 (EL2), Asp170 (EL2), His264 (EL3), Leu267 (EL3), Met270^7.35^, and Tyr271^7.36^, while the 5-substituent is located in the depth of the binding cavity in proximity to residues belonging to the TM3, TM5, and TM6 segments (Val84^3.32^, Leu85^3.33^, Thr88^3.36^, Phe168 (EL2), Met177^5.38^, Trp246^6.48^, Leu249^6.51^, His250^6.52^, Asn253^6.55^, and Ile274^7.39^).

**Figure 3:**
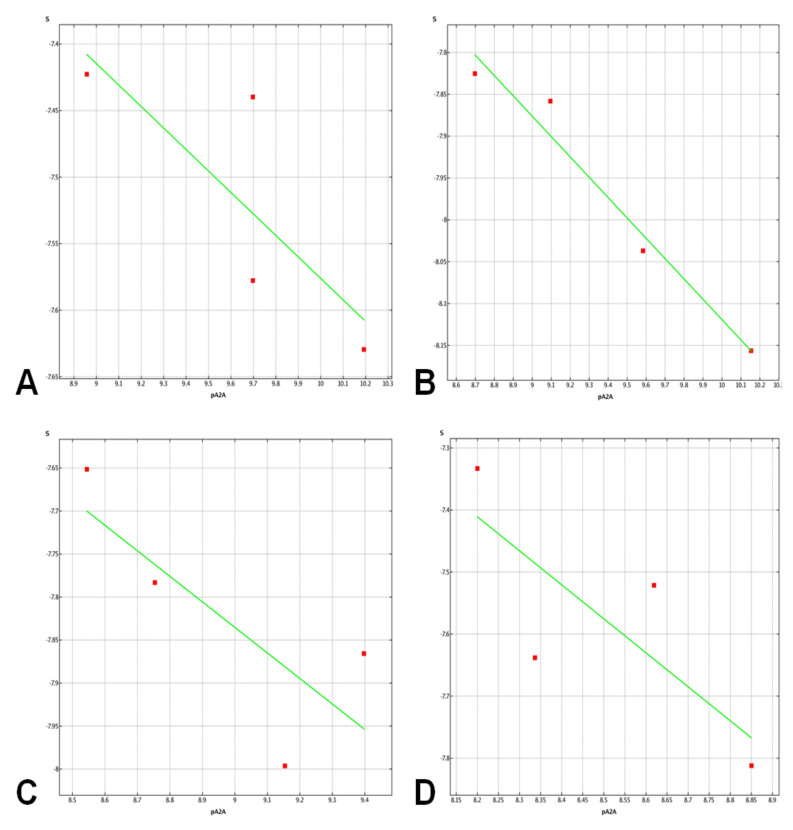
Representation of pK_i_ hA_2A_ AR vs. docking score (MOE GBVI/WSA dG score) plots for groups of compounds presenting the same 5-substituent and various 2-groups. The pK_i_ data are indicated as “pA2A”, while the docking scores are indicated as “S”. Panels (**A**–**D**) represent the plots for compounds presenting in the 5-position a furan-2yl ring, a 5-methyl-furan-2yl ring, a phenyl ring, and a 3-cyanophenyl ring, respectively. The docking tool appears quite efficient in assigning better docking scores to compounds endowed with higher hA_2A_ AR affinity.

Considering the 2-substituent, docking results suggest that this group makes mainly non-polar interactions with residues at the entrance of the binding cavity (Figure 2C). Analogous to previously reported pyrazolopyrimidine derivatives, the insertion of substituents at the 2-position of the 2-benzyl group leads to a modulation of the hA_2A_ AR affinity. The insertion of a fluorine or a chlorine atom in this position leads to the most potent compounds of the series. These groups have interaction with residues in their proximity such as Leu267, Met270^7.35^, and Tyr271^7.36^ (see Figure 2C). The effect of the various modifications at the 2-substituent appear fairly interpreted by the docking scoring function. Figure 3 shows the pK_i_ hA_2A_ AR vs. docking score (MOE GBVI/WSA dG score) plots for groups of compounds presenting the same 5-susbsituent and various 2-groups.

The 5-substituent may be an unsubstituted or a 5-methyl substituted furyl ring, or an unsubstituted or a meta-substituted phenyl ring. Compounds bearing an unsubstituted or a 5-methyl substituted furyl ring are generally endowed with the highest hA_2A_ AR affinity. This may be interpreted considering that the oxygen atom of the furyl ring gives a polar interaction with the amine function of Asn253^6.55^, even in the presence of a further methyl group at the 5-position of this substituent. In the case of the compounds bearing a 5-phenyl ring, this polar interaction is not possible and is replaced by a slightly repulsive effect between the same substituent and the above cited Asn253^6.55^. Considering the effect of the various 5-substituents on compound arrangement within the binding site, docking results show that the conformations of compounds bearing a 5-methylfuryl or a phenyl ring at the 5-position almost matched each other, even if slightly displaced compared to the conformations of the analogues bearing a 5-furyl ring. Introduction of further substituents on the 5-phenyl ring leads to a more marked rearrangement of the compounds due to steric clashes with the receptor residues.

We performed a post-docking analysis by using the *IF-E 6.0* [45] SVL script tool, which calculates the per-residue interaction energies (values in kcal/mol), where negative and positive energy values are associated with favorable and unfavorable interactions with the ligand, respectively. This tool is helpful in interpreting the binding affinities at ARs in previously reported studies [26,46,47]. To analyze the effect of the presence of a furyl ring, a 5-methylfuryl ring, or a phenyl group at the 5-position, we compared nine compounds bearing these three substituents (**8**, **18**, **4**; **9**, **19**, **5**, **6**, **15**, **1**) and the same group at the 2-position (an unsubstituted or a 2-chloro- or 2-fluoro-benzyl group). The results of this analysis are reported in Table 3.

Results show that compounds bearing a 5-methylfuryl group at the 5-position generally provide a better interaction with the binding site residues in its proximity with respect to the corresponding 5-furyl analogues, in agreement with previously observed results for pyrazolopyrimidine at the hA_2A_ AR [26]. Furthermore, compounds bearing a 5-furyl substituent afford a better interaction with the binding site residues with respect to the corresponding 5-phenyl derivatives, analogously to what was previously observed for thiazolopyrimidines at the same receptor [47]. These data are quite in agreement with the hA_2A_ AR affinity values. The significantly different interaction with Asn253^6.55^ between compounds bearing an unsubstituted or substituted furyl ring and compounds bearing a 5-phenyl group can be noticed, as above described. The only discrepancy between the interaction energy data and the affinity values was observed for compounds **18** and **19**, bearing at the 5-position a furyl or a 5-methylfuryl substituent. For these two compounds, hA_2A_ AR affinity data showed the first one as the most active, while the calculated interaction energies suggest a better interaction for the latter compound.

**Figure 4:**
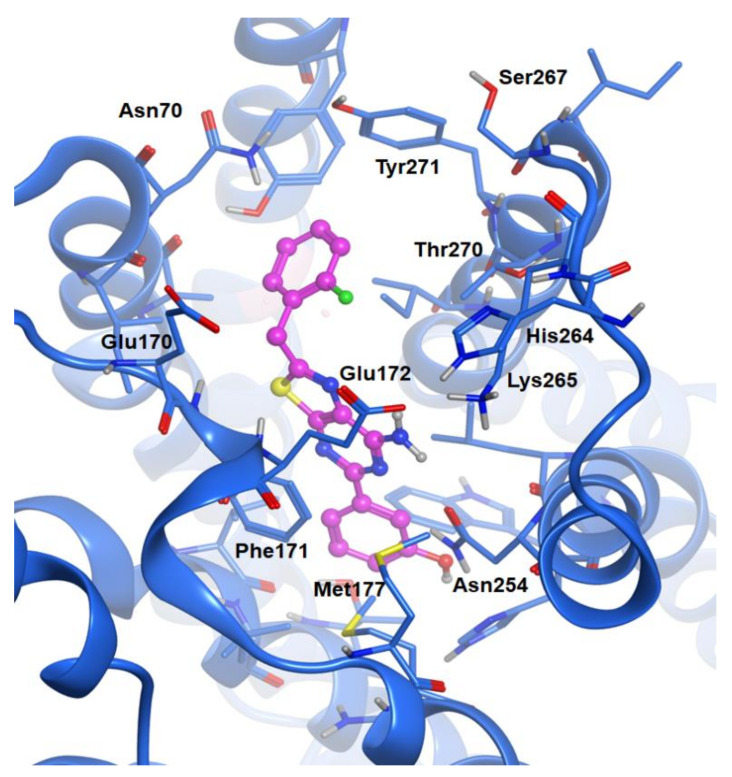
Docking-based hypothetical binding mode (top view) of the synthesized compounds at the hA_1_ AR cavity; compound **17** is represented.

Docking studies performed at the hA_1_ AR (Figure 4) show that the herein reported compounds can adopt a binding conformation highly similar to the one at the hA_2A_ AR, making analogue interactions at the two proteins (particularly in the depth of the cavity, in proximity to the 5-substituent). Slightly different environments can be observed at the entrance of the two cavities, defined by the receptor residues in the EL2 and EL3 segments. This cavity appears slightly larger at the hA_1_ AR compared with the corresponding region at the hA_2A_ AR. As a consequence, the exocyclic amine function makes a H-bond interaction with the EL2 glutamate residue at the hA_2A_ AR (Glu169), while the corresponding residue at the hA_1_ AR (Glu172) appears more distant from the same amine group. Analogously, the set of EL3 residues (Ser267, Thr270^7.35^ and Tyr271^7.36^) of hA_1_ AR in proximity to the 5-substituent makes the binding site entrance a slightly larger cavity with respect to the bulkier corresponding residues of hA_2A_ AR EL3 (Leu267, Met270^7.35^, and Tyr271^7.36^). These factors could be at the basis of the observation that the affinity data at the hA_1_ AR subtype (generally in the low nanomolar order, as indicated above) are, on average, 10-fold lower than the corresponding data at the hA_2A_ AR, even if the comparison of the data at the two receptors shows that there is a common trend.

### 2.4. Behavioral In Vivo Tests

On the basis of the results obtained in the binding and cAMP assays, we selected compound **18** for evaluation in the forced swimming test (FST) and the tail suspension test (TST) in mice, which are a widely used behavioral paradigm for the evaluation of antidepressant-like activity. Moreover, compound **18** was also tested in the sucrose preference test (SPT), which is a reward-based test highly predictive for anti-anhedonia-like activity [48,49].

#### 2.4.1. Forced Swimming Test and Tail Suspension Test

To execute the tests, mice were divided into groups, each of which consisted of 10 animals. The tests were performed after a single p.o. administration of compound **18** at different dosages (10 mg Kg^−1^ and 30 mg Kg^−1^). Amitriptyline (15 mg Kg^−1^) was used as the reference drug. Compounds were acutely administrated 26 min before the beginning of the experiment. In the FST (Table 4), the duration of immobility was recorded during the last 4 min of the 6 min test.

A decrease in the duration of immobility is indicative of an antidepressant-like effect. As shown in Table 4*,* **18** dose-dependently induced an antidepressant-like effect comparable to that induced by the clinically used drug amitriptyline. Accordingly, a similar activity was shown in the tail suspension test (Table 5) where the immobility time was measured in the first 2 min, when animals react to the inescapable stress, and in the last 4 min of the test, when the behavioral despair is established. Immobility was defined as the absence of any limb or body movements, except those caused by respiration. A reduction in the duration of the immobility time is indicative of an antidepressant-like effect.

#### 2.4.2. Sucrose Preference Test

Anhedonia, or the inability to experience pleasure, is a common symptom of depression. The mood regulatory role of **18** was studied against anhedonia evaluated in the sucrose preference test in animals treated with lipopolysaccharide (LPS), a model of neuroinflammation-induced depressive-like syndrome [49]. Compound **18** (10 mg kg^−1^ and 30 mg kg^−1^) was daily p.o administered for five consecutive days. Twenty-four hours after the last **18** treatment, LPS 1.25 mg kg^−1^ was intraperitoneally injected. Immediately after LPS injection, mice were placed in cages equipped with two bottles: a bottle of 2% sucrose solution and a bottle of water (unsweetened tap water). The consumption of the 2% sucrose solution was evaluated 6 h and 24 h after LPS injection. Amitriptyline (15 mg kg^−1^) was used as the reference drug administered following the same protocol. The preference index revealed that at 30 mg kg^−1^, **18** was able to revert the anhedonia-like behavior induced by LPS comparably to the effect of amitriptyline (Table 6).

## 3. Materials and Methods

### 3.1. Chemistry

#### 3.1.1. General Methods

Microwave-assisted syntheses were accomplished using an Initiator EXP Microwave Biotage instrument (frequency of irradiation: 2.45 GHz). Analytical silica gel plates (Merck F254), preparative silica gel plates (Merck F254, 2 mm), and silica gel 60 (Merck, 70–230 mesh) were employed for analytical and preparative TLC, and for column chromatography, respectively. All melting points were registered on a Gallenkamp melting point apparatus and resulted in being uncorrected. Elemental analyses were done with a FlashE1112 Thermofinnigan elemental analyzer for C, H, N, and the results were within ± 0.4% of the theoretical values. All final compounds showed a purity not less than 95%. Compounds were named following IUPAC rules as applied by ChemDrawUltra 9.0. The IR spectra were obtained in Nujol mulls using a Perkin-Elmer Spectrum RX I spectrometer and are expressed in cm^−1^. NMR spectra were recorded on a Bruker Avance 400 spectrometer (400 MHz for ^1^H NMR and 100 MHz for ^13^C NMR). The chemical shifts are reported in δ (ppm) and are relative to the central peak of the solvent, which was CDCl_3_ or DMSOd_6_. The following abbreviations were used: s = singlet, d = doublet, t = triplet, m = multiplet, br = broad, and ar = aromatic protons. ^1^H NMR and ^13^C APT NMR spectra of some selected derivatives (**8**, **10**, **11**, **17**, **18**) are reported in the Supporting material‎.

#### 3.1.2. General Procedure for the Synthesis of **25**–**28**

To a suspension of the 5-amino-6-sulfanylpyrimidine-2,4-diol **20** [36] (8.7 mmol) in dry NMP, the suitable arylacetyl chloride **21**, **22** [37], **23** [38], and **24** [39] (10.5 mmol) was added. The resulting mixture was heated at 150 °C for 10–15 h, then was cooled to rt and diluted with cold water (100 mL), affording a precipitate, which was collected by filtration and purified by crystallization. 2-Benzylthiazolo[5,4-d]pyrimidine-5,7-diol (**25**).

Yield: 75%. Mp: 286–289 °C (Acetic acid). ^1^H NMR (DMSO-d_6_): 4.29 (s, 2H, CH_2_), 7.29–7.35 (m, 5H, ar), 11.24 (s, 1H, OH), 11.88 (br s, 1H, OH). Anal. calcd. for (C_12_H_9_N_3_O_2_S): C, 55.59%; H, 3.50%; N, 16.21%. Anal. found: C, 55.73%; H, 3.59%; N, 16.48%. 2-(2-Chlorobenzyl)thiazolo[5,4-d]pyrimidine-5,7-diol (**26**).

Yield: 65%. Mp: >300 °C (Acetic acid/DMF). ^1^H NMR (DMSO-d_6_): 4.41 (s, 2H, CH_2_), 7.34–7.39 (m, 2H, ar), 7.49–7.52 (m, 2H, ar), 11.26 (s, 1H, OH), 11.89 (s, 1H, OH). Anal. calcd. for (C_12_H_8_ClN_3_O_2_S): C, 49.07%; H, 2.75%; N, 14.31%. Anal. found: C, 49.32%; H, 2.97%; N, 14.66%. 2-(2-Methoxybenzyl)thiazolo[5,4-d]pyrimidine-5,7-diol (**27**).

Yield: 50%. Mp: >300 °C (ethanol/Acetic acid). ^1^H NMR (DMSO-d_6_): 3.81 (s, 3H, CH_3_), 4.20 (s, 2H, CH_2_), 6.95 (t, 1H, ar, J = 7 Hz), 7.05 (d, 1H, ar, J = 8.04 Hz), 7.29–7.34 (m, 2H, ar), 11.24 (s, 1H, OH), 11.85 (s, 1H, OH). Anal. calcd. for (C_13_H_11_N_3_O_3_S): C, 53.97%; H, 3.83%; N, 14.52%. Anal. found: C, 54.28%; H, 3.07%; N, 14.66%. 2-(2-Fluorobenzyl)thiazolo[5,4-d]pyrimidine-5,7-diol (**28**).

Yield: 60%. Mp: >300 °C (Acetic acid). ^1^H NMR (DMSO-d_6_): 4.33 (s, 1H, CH_2_), 7.20–7.26 (m, 2H, ar), 7.35–7.47 (m, 2H, ar), 11.26 (s, 1H, OH), 11.90 (s, 1H, OH). Anal. calcd. for (C_12_H_8_FN_3_O_2_S): C, 51.98%; H, 2.91%; N, 15.16%. Anal. found: C, 52.12%; H, 3.15%; N, 15.33%.

#### 3.1.3. General Procedure for the Synthesis of **29**–**32**

A suspension of the 5,7-dihydroxy derivatives **25**–**28** (2.5 mmol) in POCl_3_ (10 mL) was heated at 160 °C under microwave irradiation for 30 min. The organic phase was concentrated under vacuum, then the residue was added with a mixture of ice-water (100 g) affording a precipitate that was collected by filtration and used in the next step without further purification. 2-Benzyl-5,7-dichlorothiazolo[5,4-d]pyrimidine (**29**).

Yield: 90%. ^1^H NMR (DMSO-d_6_): 4.62 (s, 2H, CH_2_), 7.34–7.43 (m, 5H, ar). 5,7-Dichloro-2-(2-chlorobenzyl)thiazolo[5,4-d]pyrimidine (**30**).

Yield: 65%. ^1^H NMR (DMSO-d_6_): 4.73 (s, 2H, CH_2_), 7.41–7.43 (m, 2H, ar), 7.54–7.56 (m, 1H, ar), 7.60–7.62 (m, 1H, ar). 5,7-Dichloro-2-(2-methoxybenzyl)thiazolo[5,4-d]pyrimidine (**31**).

Yield: 90%. ^1^H NMR (DMSO-d_6_): 3.73 (s, 3H, OCH_3_), 4.49 (s, 2H, CH_2_), 6.98 (t, 1H, ar, J = 7.36 Hz), 7.08 (d, 1H, ar, J = 8.16 Hz), 7.34–7.41 (m, 2H, ar). 5,7-Dichloro-2-(2-fluorobenzyl)thiazolo[5,4-d]pyrimidine (**32**).

Yield: 85%. ^1^H NMR (DMSO-d_6_): 4.66 (s, 2H, CH_2_), 7.26–7.27 (m, 2H, ar), 7.42–7.55 (m, 2H, ar).

#### 3.1.4. General Procedure for the Synthesis of **33**–**36**

A suspension of the 5,7-dichloro derivatives **29–32** (5 mmol) in a mixture of 33% aqueous ammonia solution (20 mL) and ethanol (15 mL) was heated at reflux for 6 h. The reaction mixture was then cooled to rt, affording a solid, which was collected by filtration. 2-Benzyl-5-chlorothiazolo[5,4-d]pyrimidin-7-amine (**33**).

Yield: 65%. Mp: 194-197 °C (ethanol). ^1^H NMR (DMSO-d_6_): 4.45 (s, 2H, CH_2_), 7.31–7.39 (m, 5H, ar), 8.16 (br s, 2H, NH_2_). IR: 3453, 3262. Anal. calcd. for (C_12_H_9_ClN_4_S): C, 52.08%; H, 3.28%; N, 20.24%. Anal. found: C, 52.33%; H, 3.51%; N, 20.44%. 5-Chloro-2-(2-chlorobenzyl)thiazolo[5,4-d]pyrimidin-7-amine (**34**).

Yield: 77%. Mp: 186–189 °C (column chromatography, eluting system: ethyl acetate/cyclohexane 5/5). ^1^H NMR (DMSO-d_6_): 4.56 (s, 1H, CH_2_), 7.39–7.41 (m, 2H, ar), 7.53 (d, 2H, ar, J = 7.8 Hz), 8.14 (br s, 2H, NH_2_). IR: 3445, 3273. Anal. calcd. for (C_12_H_8_Cl_2_N_4_S): C, 46.32%; H, 2.59%; N, 18.00%. Anal. found: C, 46.70%; H, 2.88%; N, 17.93%. 5-Chloro-2-(2-methoxybenzyl)thiazolo[5,4-d]pyrimidin-7-amine (**35**).

Yield: 55%. Mp: 210–213 °C (acetic acid). ^1^H NMR (DMSO-d_6_): 3.73 (s, 3H, CH_3_), 6.97 (t, 1H, ar, J = 7.36 Hz), 7.07 (d, 1H, ar, J = 8.48 Hz), 7.32–7.36 (m, 2H, ar), 8.14 (br s, 2H, NH_2_). IR: 3437, 3277. Anal. calcd. for (C_13_H_11_ClN_4_OS): C, 50.90%; H, 3.61%; N, 18.26%. Anal. found: C, 51.21%; H, 3.86%; N, 18.39%. 5-Chloro-2-(2-fluorobenzyl)thiazolo[5,4-d]pyrimidin-7-amine (**36**).

Yield: 85%. Mp: 193–195 °C (ethyl acetate). ^1^H NMR (DMSO-d_6_): 7.21–7.28 (m, 2H, ar), 7.37–7.42 (m, 1H, ar), 7.47–7.50 (m, 1H, ar), 8.15 (s, 2H, NH_2_). IR: 3306, 3159. Anal. calcd. for (C_12_H_8_ClFN_4_S): C, 48.90%; H, 2.74%; N, 19.01%. Anal. found: C, 48.66%; H, 2.99%; N, 18.75%.

#### 3.1.5. General Procedure for the Synthesis of **1**–**19**

To a suspension of the 5-chloro-derivatives **33**–**36** (1 mmol) in dimethoxyethane (8.5 mL) and water (2.0 mL), the suitable boronic acids (3 mmol), tetrakis (0.1 mmol), and Na_2_CO_3_ (10 mmol) were added. The mixture was refluxed for 4 h under a N_2_ atmosphere (compounds **1**–**5**, **9**), or microwave irradiated at 160 °C for 30 min (compounds **6**–**8**, **10**–**19**). The suspension was treated with water (150 mL) and if a solid was formed (compounds **5**–**6**, **8**–**9**, **11**, **13**–**15**, **18**–**19**), it was collected by filtration and washed with water. Alternatively, the solution was extracted with ethyl acetate (40 mL × 3), and the organic phases were collected and dried with Na_2_SO_4._ Then, the solvent was evaporated to yield a solid which, after treatment with diethyl ether, was collected by filtration compounds **1**–**4**, **7**, **10**, **12**, **16**–**17**). The crude products were purified by chromatography and/or crystallization as specified below. 2-Benzyl-5-phenylthiazolo[5,4-d]pyrimidin-7-amine (**1**).

Yield: 20%. Mp: 171–173 °C (column chromatography, eluting system: ethyl acetate 3/cyclohexane 7). ^1^H NMR (DMSO-d_6_): 4.46 (s, 2H, CH_2_), 7.30–7.33 (m, 1H, ar), 7.37–7.42 (m, 4H, ar), 7.46–7.48 (m, 3H, ar), 7.64 (br s, 2H, NH_2_), 8.31–8.34 (m, 2H, ar). Anal. calcd. for (C_18_H_14_N_4_S): C, 67.90%; H, 4.43%; N, 17.60%. Anal. found: C, 67.63%; H, 4.72%; N, 17.95%. 3-(7-Amino-2-benzylthiazolo[5,4-d]pyrimidin-5-yl)benzonitrile (**2**).

Yield: 74%. Mp: 175–178 °C (column chromatography, eluting system: ethyl acetate 4/cyclohexane 6). ^1^H NMR (DMSO-d_6_): 4.48 (s, 2H, CH_2_), 7.29–7.33 (m, 1H, ar), 7.37–7.43 (m, 4H, ar), 7.69–7.73 (m, 1H, ar), 7.82 (br s, 2H, NH_2_), 7.94–7.96 (m, 1H, ar), 8.59–8.60 (m, 2H, ar). Anal. calcd. for (C_19_H_13_N_5_S): C, 66.45%; H, 3.82%; N, 20.39%. Anal. found: C, 66.58%; H, 4.03%; N, 20.61%. 3-(7-Amino-2-benzylthiazolo[5,4-d]pyrimidin-5-yl)phenol (**3**).

Yield: 55%. Mp: 179–181 °C (column chromatography, eluting system: ethyl acetate 4/cyclohexane 6). ^1^H NMR (DMSO-d_6_): 4.46 (s, 2H, CH_2_), 6.84–6.86 (m, 1H, ar), 7.24 (t, 1H, ar, J = 7.6 Hz), 7.27–7.31 (m, 1H, ar), 7.37–7.40 (m, 4H, ar), 7.62 (br s, 2H, NH_2_), 7.77–7.79 (m, 2H, ar), 9.49 (s, 1H, OH). Anal. calcd. for (C_18_H_14_N_4_OS): C, 64.65%; H, 4.22%; N, 16.75%. Anal. found: C, 64.87%; H, 4.45%; N, 17.07%. 2-Benzyl-5-(furan-2-yl)thiazolo[5,4-d]pyrimidin-7-amine (**4**).

Yield: 60%. Mp: 194–197 °C (column chromatography, eluting system: ethyl acetate 5/cyclohexane 5). ^1^H NMR (DMSO-d_6_): 4.44 (s, 2H, CH_2_), 6.63–6.64 (m, 1H, ar), 7.13–7.14 (m, 1H, ar), 7.31–7.41 (m, 5H, ar), 7.70 (br s, 2H, NH_2_), 7.82–7.83 (m, 1H, ar). Anal. calcd. for (C_16_H_12_N_4_OS): C, 62.32%; H, 3.92%; N, 18.17%. Anal. found: C, 62.65%; H, 4.15%; N, 18.23%. 2-Benzyl-5-(5-methylfuran-2-yl)thiazolo[5,4-d]pyrimidin-7-amine (**5**).

Yield: 75%. Mp: 205–208 °C (column chromatography, eluting system: ethyl acetate 5/cyclohexane 5). ^1^H NMR (DMSO-d_6_): 2.36 (s, 3H, CH_3_), 4.43 (s, 2H, CH_2_), 6.25–6.26 (m, 1H, ar), 7.03–7.04 (m, 1H, ar), 7.29–7.39 (m, 5H, ar), 7.66 (br s, 2H, NH_2_). Anal. calcd. for (C_17_H_14_N_4_OS): C, 63.33%; H, 4.38%; N, 17.38%. Anal. found: C, 62.99%; H, 4.42%; N, 17.42%. 2-(2-Chlorobenzyl)-5-phenylthiazolo[5,4-d]pyrimidin-7-amine (**6**).

Yield: 25%. Mp: 155–158 °C (column chromatography, eluting system: ethyl acetate 3/cyclohexane 7). ^1^H NMR (DMSO-d_6_): 4.58 (s, 2H, CH_2_), 7.39–7.42 (m, 2H, ar), 7.46–7.48 (m, 3H, ar,), 7.53–7.58 (m, 2H, ar), 7.66 (br s, 2H, NH_2_), 8.31–8.33 (m, 2H, ar). ^13^C-NMR (DMSO-d_6_): 38.23, 128.25, 128.28, 128.78, 129.03, 130.06, 130.16, 130.63, 132.35, 133.95, 135.31, 137.94, 157.08, 159.87, 164.19, 165.32. Anal. calcd. for (C_18_H_13_ClN_4_S): C, 61.27%; H, 3.71%; N, 15.88%. Anal. found: C, 61.44%; H, 3.97%; N, 16.03%. 3-(7-Amino-2-(2-chlorobenzyl)thiazolo[5,4-d]pyrimidin-5-yl)benzonitrile (**7**).

Yield: 35%. Mp: 171–174 °C (column chromatography, eluting system: ethyl acetate 4/cyclohexane 6). ^1^H NMR (DMSO-d_6_): 4.60 (s, 2H, CH_2_), 7.40–7.42 (m, 2H, ar), 7.54–7.59 (m, 2H, ar), 7.71 (t, 1H, ar, J = 7.8 Hz), 7.82 (br s, 2H, NH_2_), 7.95 (d, 1H, ar, J = 7.6 Hz), 8.59–8.63 (m, 2H, ar). ^13^C-NMR (DMSO-d_6_): 38.26, 112.08, 114.62, 119.09, 128.31, 129.45, 130.10, 130.17, 130.35, 131.61, 132.38, 132.63, 133.97, 135.22, 139.06, 145.71, 157.15, 157.78, 163.98, 166.20. IR: 3483, 3375, 2228. Anal. calcd. for (C_19_H_12_ClN_5_S): C, 60.40%; H, 3.20%; N, 18.53%. Anal. found: C, 60.56%; H, 3.33%; N, 18.71%. 2-(2-Chlorobenzyl)-5-(furan-2-yl)thiazolo[5,4-d]pyrimidin-7-amine (**8**).

Yield: 27%. Mp: 187–189 °C (column chromatography, eluting system: ethyl acetate 4/cyclohexane 6). ^1^H NMR (DMSO-d_6_): 4.56 (s, 2H, CH_2_), 6.63–6.64 (m, 1H, ar), 7.13–7.14 (m,1H, ar), 7.39–7.41 (m, 2H, ar), 7.52–7.57 (m, 2H, ar), 7.69 (s, 2H, NH_2_), 7.83 (s, 1H, ar). ^13^C-NMR (DMSO-d_6_): 38.19, 112.60, 112.90, 128.28, 128.78, 130.05, 130.15, 132.38, 133.94, 135.26, 145.45, 152.51, 153.40, 157.06, 163.70, 165.10. IR: 3314, 3175. Anal. calcd. for (C_16_H_11_ClN_4_OS): C, 56.06%; H, 3.23%; N, 16.34%. Anal. found: C, 56.15%; H, 3.13%; N, 16.41%. 2-(2-Chlorobenzyl)-5-(5-methylfuran-2-yl)thiazolo[5,4-d]pyrimidin-7-amine (**9**).

Yield: 20%. Mp: 201–203 °C (column chromatography, eluting system: ethyl acetate 4/cyclohexane 6). ^1^H NMR (DMSO-d_6_): 2.35 (s, 3H, CH_3_), 4.55 (s, 2H, CH_2_), 6.25–6.26 (m, 1H, ar), 7.02–7.03 (m, 1H, ar), 7.39–7.41 (m, 2H, ar), 7.52–7.57 (m, 2H, ar), 7.66 (br s, 2H, NH_2_). IR: 3350, 3302. Anal. calcd. for (C_17_H_13_ClN_4_OS): C, 57.22%; H, 3.67%; N, 15.70%. Anal. found: C, 57.50%; H, 3.73%; N, 15.59%. 2-(2-Methoxybenzyl)-5-phenylthiazolo[5,4-d]pyrimidin-7-amine (**10**).

Yield: 20%. Mp: 158–160 °C (column chromatography, eluting system: ethyl acetate 4/cyclohexane 6). ^1^H NMR (DMSO-d_6_): 3.82 (s, 3H, CH_3_), 4.37 (s, 2H, CH_2_), 6.98 (t, 1H, ar, J = 7.4 Hz), 7.08 (d, 1H, ar, J = 8.4 Hz), 7.32–7.36 (m, 2H, ar), 7.45–7.47 (m, 3H, ar), 7.64 (br s, 2H, NH_2_), 8.30–8.33 (m, 2H, ar). ^13^C-NMR (DMSO-d_6_): 35.40, 55.91, 111.73, 121.16, 125.54, 128.24, 128.76, 128.86, 129.65, 130.54, 131.15, 138.01, 156.97, 157.50, 159.67, 164.14, 167.01. IR: 3441, 3292. Anal. calcd. for (C_19_H_16_N_4_OS): C, 65.50%; H, 4.63%; N, 16.08%. Anal. found: C, 65.59%; H, 4.44%; N, 16.26%. 3-(7-Amino-2-(2-methoxybenzyl)thiazolo[5,4-d]pyrimidin-5-yl)benzonitrile (**11**).

Yield: 25%. Mp: 205–207 °C (column chromatography, eluting system: ethyl acetate 4/cyclohexane 6). ^1^H NMR (DMSO-d_6_): 3.82 (s, 3H, CH_3_), 4.38 (s, 2H, CH_2_), 6.98 (t, 1H, ar, J = 7.4 Hz), 7.08 (d, 1H, ar, J = 8.4 Hz), 7.33–7.36 (m, 2H, ar), 7.70 (t, 1H, ar, J = 7.8 Hz), 7.78 (br s, 2H, NH_2_), 7.94 (d, 1H, ar, J = 7.7 Hz), 8.59–8.63 (m, 2H, ar). ^13^C-NMR (DMSO-d_6_): 35.45, 55.88, 111.73, 112.06, 119.10, 121.15, 125.45, 129.28, 129.70, 130.31, 131.16, 131.57, 132.57, 133.87, 139.13, 157.04, 157.51, 157.57, 163.94, 167.89. IR: 3449, 3339, 2226. Anal. calcd. for (C_20_H_15_N_5_OS): C, 64.33%; H, 4.05%; N, 18.75%. Anal. found: C, 64.55%; H, 3.86%; N, 18.82%. 3-(7-Amino-2-(2-methoxybenzyl)thiazolo[5,4-d]pyrimidin-5-yl)phenol (**12**).

Yield: 33%. Mp: 123–126 °C (column chromatography, eluting system: ethyl acetate 4/cyclohexane 6). ^1^H NMR (DMSO-d_6_): 3.81 (s, 3H, CH_3_), 4.36 (s, 2H, CH_2_), 6.83–6.85 (m, 1H, ar), 6.98 (t, 1H, ar, J = 7.4 Hz), 7.07 (d, 1H, ar, J = 8.5 Hz), 7.22–7.36 (m, 3H, ar), 7.59 (br s, 2H, NH_2_), 7.75–7.76 (m, 2H, ar), 9.50 (s, 1H, OH). ^13^C-NMR (DMSO-d_6_): 35.39, 55.88, 111.72, 115.11, 117.61, 119.19, 121.11, 125.53, 128.81, 129.64, 131.15, 139.42, 156.88, 157.51, 157.83, 159.76, 164.09, 166.92. IR: 3327, 3165. Anal. calcd. for (C_19_H_16_N_4_O_2_S): C, 62.62%; H, 4.43%; N, 15.37%. Anal. found: C, 62.48%; H, 4.58%; N, 15.49%. 5-(Furan-2-yl)-2-(2-methoxybenzyl)thiazolo[5,4-d]pyrimidin-7-amine (**13**).

Yield: 20%. Mp: 189–192 °C (column chromatography, eluting system: ethyl acetate 4/cyclohexane 6). ^1^H NMR (DMSO-d_6_): 3.81 (s, 3H, CH_3_), 4.35 (s, 2H, CH_2_), 6.63–6.64 (m, 1H, ar), 6.97 (t, 1H, ar, J = 7.4 Hz), 7.06–7.13 (m, 2H, ar), 7.32–7.36 (m, 2H, ar), 7.67 (br s, 2H, NH_2_), 7.82 (s, 1H, ar). IR: 3283, 3123. Anal. calcd. for (C_17_H_14_N_4_O_2_S): C, 60.34%; H, 4.17%; N, 16.56%. Anal. found: C, 60.63%; H, 4.29%; N, 16.71%. 2-(2-Methoxybenzyl)-5-(5-methylfuran-2-yl)thiazolo[5,4-d]pyrimidin-7-amine (**14**).

Yield: 24%. Mp: 197–199 °C (column chromatography, eluting system: ethyl acetate 4/cyclohexane 6). ^1^H NMR (DMSO-d_6_): 3.80 (s, 3H, CH_3_), 4.34 (s, 2H CH_2_), 6.25–6.26 (m, 1H, ar), 6.95–7.08 (m, 3H, ar), 7.32–7.35 (m, 2H, ar), 7.62 (br s, 2H, NH_2_). ^13^C-NMR (DMSO-d_6_): 35.33, 55.87, 109.00, 111.72, 113.91, 121.11, 125.53, 128.35, 129.63, 131.12, 151.06, 153.26, 154.49, 156.92, 157.49, 163.71, 166.35. IR: 3315, 3172. Anal. calcd. for (C_18_H_16_N_4_O_2_S): C, 61.35%; H, 4.58%; N, 15.90%. Anal. found: C, 61.47%; H, 4.44%; N, 16.11%. 2-(2-Fluorobenzyl)-5-phenylthiazolo[5,4-d]pyrimidin-7-amine (**15**).

Yield: 45%. Mp: 192–195 °C (column chromatography, eluting system: ethyl acetate 5/cyclohexane 5). ^1^H NMR (DMSO-d_6_): 4.50 (s, 2H, CH_2_), 7.23–7.29 (m, 2H, ar), 7.38–7.53 (m, 5H, ar), 7.68 (br s, 2H, NH_2_), 8.31–8.33 (m, 2H, ar). IR: 3456, 3273, 3140. Anal. calcd. for (C_18_H_13_FN_4_S): C, 64.27%; H, 3.90%; N, 16.66%. Anal. found: C, 64.33%; H, 4.07%; N, 16.74%. 3-(7-Amino-2-(2-fluorobenzyl)thiazolo[5,4-d]pyrimidin-5-yl)benzonitrile (**16**).

Yield: 20%. Mp: 152–154 °C (column chromatography, eluting system: ethyl acetate 4/cyclohexane 6). ^1^H NMR (DMSO-d_6_): 4.52 (s, 2H, CH_2_), 7.23–7.29 (m, 2H, ar), 7.38–7.44 (m, 1H, ar), 7.49–7.53 (m, 1H, ar) 7.71 (t, 1H, ar, J = 7.8 Hz), 7.81 (br s, 2H, NH_2_), 7.95 (d, 1H, ar, J = 7.6 Hz), 8.60–8.63 (m, 2H, ar). IR: 3504, 3331, 2233. Anal. calcd. for (C_19_H_12_FN_5_S): C, 63.15%; H, 3.35%; N, 19.38%. Anal. found: C, 63.31%; H, 3.49%; N, 19.47%. 3-(7-Amino-2-(2-fluorobenzyl)thiazolo[5,4-d]pyrimidin-5-yl)phenol (**17**).

Yield: 35%. Mp: 172–175 °C (column chromatography, eluting system: ethyl acetate 5/cyclohexane 5). ^1^H NMR (DMSO-d_6_): 4.49 (s, 2H, CH_2_), 6.84–6.86 (m, 1H, ar), 7.23–7.28 (m, 3H, ar), 7.38–7.43 (m, 1H, ar), 7.50 (t, 1H, ar, J = 7.6Hz), 7.59 (br s, 2H, NH_2_), 7.75–7.77 (m, 2H, ar), 9.48 (s, 1H, OH). ^13^C-NMR (DMSO-d_6_): 35.36, 55.88, 111.71, 112.62, 112.70, 121.13, 125.48, 128.58, 129.67, 131.15, 152.55, 153.22, 156.94, 157.49, 163.62, 166.83. Anal. calcd. for (C_18_H_13_FN_4_OS): C, 61.35%; H, 3.72%; N, 15.90%. Anal. found: C, 61.42%; H, 3.59%; N, 16.09%. 2-(2-Fluorobenzyl)-5-(furan-2-yl)thiazolo[5,4-d]pyrimidin-7-amine (**18**).

Yield: 35%. Mp: 173–175 °C (column chromatography, eluting system: ethyl acetate 4/cyclohexane 6/methanol 1). ^1^H NMR (DMSO-d_6_): 4.48 (s, 2H, CH_2_), 6.63–6.64 (m, 1H, ar), 7.14 (d, 1H, ar, J = 3.2 Hz), 7.22–7.28 (m, 2H, ar), 7.38–7.43 (m, 1H, ar), 7.50 (t, 1H, ar, J = 7.6 Hz), 7.67 (br s, 2H, NH_2_), 7.83 (s, 1H, ar). IR: 3310, 3134. Anal. calcd. for (C_16_H_11_FN_4_OS): C, 58.89%; H, 3.40%; N, 17.17%. Anal. found: C, 59.05%; H, 3.47%; N, 17.36%. 2-(2-Fluorobenzyl)-5-(5-methylfuran-2-yl)thiazolo[5,4-d]pyrimidin-7-amine (**19**).

Yield: 24%. Mp: 186–188 °C (column chromatography, eluting system: ethyl acetate 4/cyclohexane 6). ^1^H NMR (DMSO-d_6_): 2.35 (s, 3H, CH_3_), 4.47 (s, 2H, CH_2_), 6.26 (s, 1H, ar), 7.03 (s, 1H, ar), 7.22–7.28 (m, 2H, ar), 7.37–7.42 (m, 1H, ar), 7.49 (t, 1H, ar, J = 7.6 Hz), 7.65 (br s, 2H, NH_2_). ^13^C-NMR (DMSO-d_6_): 14.06, 33.73, 109.05, 114.10, 115.96, 116.17, 124.50, 124.65, 125.33, 125.36, 128.45, 130.20, 130.28, 132.06, 132.09, 150.97, 153.42, 154.61, 157.04, 159.65, 162.09, 163.84, 164.63. IR: 3491, 3286. Anal. calcd. for (C_17_H_13_FN_4_OS): C, 59.99%; H, 3.85%; N, 16.46%. Anal. found: C, 56.15%; H, 3.97%; N, 16.55%.

### 3.2. Pharmacological Assays

#### 3.2.1. Membrane Preparation

CHO cells, stably expressing *h*ARs, were grown adherently and maintained in Dulbecco’s modified Eagle’s medium (DMEM) with nutrient mixture F12 supplemented with 10% fetal bovine serum (FBS), penicillin (100 µg/mL), streptomycin (100 µg/mL), sodium pyruvate (1 mM), and geneticin (0.1 mg/mL). Cells were maintained in a humidified incubator containing 5% CO_2_ and 95% air.

Membranes were prepared as previously reported [34]. Briefly, cells were homogenized in cold lysis buffer (5 mM Tris HCl, 2 mM EDTA, pH 7.4), centrifuged at low speed spin for 10 min, and subsequently, the supernatant was spun at 37,000 rpm. Membrane pellet was resuspended in the specific buffer and stored at −80 °C.

#### 3.2.2. Radioligand Binding

The binding affinity of the novel compounds was evaluated using radioligand competition experiments in CHO cells stably transfected with *h*A_1_ AR, *h*A_2_A AR, and *h*A_3_ AR subtypes. The radioligands used were 1 nM [^3^H] CCPA for hA_1_ AR (K_D_ = 1.1 nM), 10 nM [^3^H] NECA for hA_2A_ AR (K_D_ = 20 nM); 1 nM [^3^H] HEMADO for A_3_ AR (K_D_ = 1.5 nM). The potency at *h*A_2B_ AR, expressed on CHO cells, was determined by inhibition of NECA-stimulated adenylyl cyclase activity [34].

#### 3.2.3. GloSensor cAMP Assay

The intrinsic activity of understudy compounds was evaluated through the GloSensor cAMP assay, as described previously [50]. Briefly, cells stably expressing the *h*A_1_, *h*A_2A_, and *h*A_2B_ ARs and the biosensor were incubated for 2 h at rt in equilibration medium containing 3% *v*/*v* GloSensor cAMP reagent stock solution, 10% FBS, and 87% CO_2_ independent medium. Afterward, cells were dispensed in the wells of a 384-well plate and the reference agonist NECA or the compounds, at different concentrations, were tested. The antagonist profile of compounds was evaluated by assessing their ability to counteract NECA-induced increase (A_2A_ and A_2B_ ARs) or decrease (A_1_ AR) of cAMP accumulation [51]. Responses were expressed as percentage of the maximal relative luminescence units (RLU).

#### 3.2.4. Statistical Analysis

Binding data and concentration–response curves were fitted by a nonlinear regression with the Prism program (GraphPAD Prism 7 Software, San Diego, CA, USA). Each concentration was tested 3–5 times in duplicate and the K_i_ or IC_50_ (the concentration of antagonists that produces 50% inhibition of the agonist effect) values are given as the mean ± standard error (S.E.).

### 3.3. In Vivo Assays

#### 3.3.1. Animals

Male CD-1 albino mice (22–25 g; Envigo, Varese, Italy) were housed in CeSAL (Centro Stabulazione Animali da Laboratorio, University of Florence, Florence, Italy) and used at least one week after their arrival. Animals were housed in 26 cm × 41 cm cages (12 mice each) and fed with a standard laboratory diet and tap water ad libitum. They were kept at 23 ± 1 °C with a 12 h light/dark cycle, light at 7 a.m. Compound **18** was administered per o.s. whereas amitriptyline was by a s.c. route following the most widely used approach reported in the literature.

#### 3.3.2. Forced Swimming Test

Mice were individually placed into glass cylinders (height: 25 cm, diameter: 10 cm) containing 12 cm of water maintained at 22–23 °C for 6 min. Immobility was considered the animal floated in the water, in an upright position, and made only small movements to keep its head above water. The immobility time was recorded during the last 4-min of the 6-min test. A decrease in the duration of immobility is indicative of an antidepressant-like effect [52].

#### 3.3.3. Tail Suspension Test

A piece of tape was adhered to the upper middle of the tail of each animal, creating a flap with the overlap of tape. Mice were suspended from a plastic rod mounted 50 cm above the surface by fastening the tail to the rod with adhesive tape. The duration of the test was 6 min and the immobility time was measured in the first 2 min, when animals react to the inescapable stress, and in the last 4 min of the test, when the behavioral despair is established. Immobility was defined as the absence of any limb or body movements, except those caused by respiration.

#### 3.3.4. LPS-Induced Anhedonia

Lipopolysaccharide (LPS) from *Escherichia coli* was purchased from Sigma-Aldrich, (Milan, Italy) freshly dissolved in sterile saline, and injected intraperitoneally (i.p.) at the dose of 1.25 mg kg^−1^ [49] after five consecutive days of treatment with compound **18**, 1 h after the last administration of the compound. Behavioral tests were performed before 6 h and 24 h after LPS administration.

#### 3.3.5. Sucrose Preference Test

Mice were placed in cages equipped with a couple of bottles, one containing 2% sucrose solution, the second with water (unsweetened tap water). The consumption of the 2% sucrose solution was evaluated 6 h and 24 h after the beginning of the experiment. The preference index was calculated according to the following formula: preference index = volume consumed sucrose solution/(volume consumed sucrose solution + volume consumed water).

#### 3.3.6. Statistical Analysis

Behavioral tests were performed by visual observation by researchers blinded to the treatments. Results were expressed as means ± SEM, variance was analyzed by ANOVA. A Bonferroni’s significant difference procedure was used as post-hoc comparison. *p* values of less than 0.05 and 0.01 were considered significant. Data were analyzed using the “Origin 8.1” software.

### 3.4. Molecular Modeling Studies

#### 3.4.1. Refinement of the Human A_2A_ AR and A_1_ AR Structures

The crystal structure of the hA_2A_ AR and hA_1_ AR in complex with ZM241385 (pdb code: 5NM4; 1.7-Å resolution [40]) and PSB36 (pdb code: 5N2S; 3.3-Å resolution [44]), respectively, were selected as molecular targets for molecular docking experiments.

The receptor structures were checked with the Homology Modeling tool of MOE [41], by correcting the amino acidic sequence (due to the presence of some mutations and external segments within the crystallized thermostabilized receptor) to restore the wild type primary structure and by adding and energetically minimizing the hydrogen atoms.

#### 3.4.2. Molecular Docking Analysis

All compound structures were docked into the hA_2A_ AR and hA_1_ AR binding site using three docking tools: the Induced Fit docking protocol of MOE [41], the genetic algorithm docking tool of CCDC Gold [42], and the docking tool of Cresset Flare [43]. The Induced Fit docking protocol of MOE is divided into the following stages: conformational analysis of ligands; placement; scoring; induced fit; and rescoring. Alpha HB scoring function was employed in this task. Gold tool was used with default efficiency settings through MOE interface by selecting Chemscore as scoring function. Flare docking tool was used with “accurate but slow” settings and “extra precision” quality.

#### 3.4.3. Post Docking Analysis. Residue Interaction Analysis

The ligand–target interactions were analyzed by using the *IF-E 6.0* SVL script tool [45], which calculates and displays the residue interaction forces as 3D vectors and calculates the per-residue interaction energies (negative and positive energy values being associated to favorable and unfavorable interactions, respectively). A shell of A_2A_ AR residues within a 10 Ǻ distance from the ligand were considered for this analysis.

## 4. Conclusions

This work has led to the identification of a new set of extremely potent hA_1_/hA_2A_ AR dual antagonists belonging to the 7-aminothiazolo[5,4-d]pyrimidine series. Most of the new derivatives were endowed with nanomolar and subnanomolar affinity values for the hA_1_ and hA_2A_, respectively, and high selectivity versus the other ARs. The best combined activity and selectivity at the A_1_/A_2A_ ARs was shown by derivative **18**, which was chosen to evaluate its antidepressant-like activity. Thus, **18** was tested in in vivo models of depression (i.e., the FST and the TST), showing an efficacy comparable to that of the clinically used drug amitriptyline. Compound **18** was also tested in the sucrose preference test to evaluate its anti-anhedonia-like effect. Interestingly, **18** showed a good anti-anhedonia-like activity comparable to that of amitriptyline.

To conclude, we identified new potent hA_1_/hA_2A_ AR dual antagonists belonging to the 7-amino-thiazolo[5,4-d]pyrimidine series, which can be considered as promising candidates for further pharmacological evaluation as antidepressant agents also able to contrast anhedonia.

## Figures and Tables

**Table 1:**
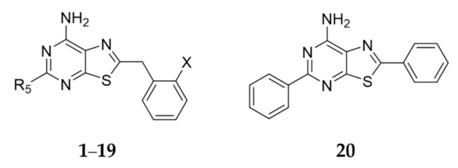
Binding affinities (K_i_) at hA_1_, hA_2A_, and hA_3_ ARs and potencies (IC_50_) at hA_2B_ ARs.

	X	R_5_	hA_1_K_i_ (nM) ^a^	hA_2A_K_i_ (nM) ^b^	hA_3_K_i_ (nM) ^c^	hA_2B_IC_50_ (nM) ^d^
**1**	H	C_6_H_5_	20 ± 4.3	2.9 ± 0.04	26.6 ± 6.0	>30,000
**2**	H	C_6_H_4_-3-CN	51.7 ± 11.7	4.6 ± 0.9	76 ± 0.9	>30,000
**3**	H	C_6_H_4_-3-OH	15.7 ± 3.4	7.3 ± 1.0	103.6 ± 21.1	>30,000
**4**	H	furan-2yl	15.5 ± 3.6	1.1 ± 0.2	65.4 ± 12	794 ± 196
**5**	H	furan-2yl-5-CH_3_	6.8 ± 1.2	0.8 ± 0.04	18.8 ± 1.9	1096 ± 228
**6**	Cl	C_6_H_5_	2.8 ± 0.05	0.4 ± 0.04	143 ± 0.2	>30,000
**7**	Cl	C_6_H_4_-3-CN	72.8 ± 15	6.3 ± 0.18	295.1 ± 0.6	6785 ± 62
**8**	Cl	furan-2yl	3.8 ± 0.6	0.2 ± 0.03	4786 ± 5.1	1887 ± 175
**9**	Cl	furan-2yl-5-CH_3_	0.5 ± 0.1	0.07 ± 0.006	8.5 ± 1.6	8847 ± 1445
**10**	OCH_3_	C_6_H_5_	4.5 ± 0.8	0.7 ± 0.1	37.4 ± 1	>30,000
**11**	OCH_3_	C_6_H_4_-3-CN	28.4 ± 4	2.4 ± 0.09	54.2 ± 1.2	1536 ± 35
**12**	OCH_3_	C_6_H_4_-3-OH	10.2 ± 0.3	1.9 ± 0.4	79.6 ± 2.3	7424 ± 876
**13**	OCH_3_	furan-2yl	2.4 ± 0.1	0.2 ± 0.03	48.6 ± 2.4	1536 ± 35
**14**	OCH_3_	furan-2yl-5-CH_3_	11.5 ± 2.8	2.0 ± 0.6	18.2 ± 5	2416 ± 545
**15**	F	C_6_H_5_	1.9 ± 0.05	1.8 ± 0.6	72.1 ± 5.3	>30,000
**16**	F	C_6_H_4_-3-CN	21.5 ± 5.1	1.4 ± 0.3	95.5 ± 1.5	1514 ± 121
**17**	F	C_6_H_4_-3-OH	0.5 ± 0.1	13.8 ± 0.06	455.9 ± 15	3237 ± 136
**18**	F	furan-2yl	1.9 ± 0.08	0.06 ± 0.02	93.1 ± 2.8	384 ± 55
**19**	F	furan-2yl-5-CH_3_	5.7 ± 0.2	0.26 ± 0.01	50.2 ± 9.2	1695 ± 228
**20** ^e^			148 ± 16	19 ± 6.2	84 ± 13	>30,000

^a^ Displacement of specific [^3^H]-CCPA binding at human A_1_ AR expressed in CHO cells, (*n* = 3–6). ^b^ Displacement of specific [^3^H]-NECA binding at human A_2A_ AR expressed in CHO cells. ^c^ Displacement of specific [^3^H]-HEMADO binding at hA_3_ AR expressed in CHO cells. ^d^ IC_50_ values of the inhibition of NECA-stimulated adenylyl cyclase activity in CHO cells expressing hA_2B_ AR. Data are expressed as means ± SEM. ^e^ Reference [34].

**Table 2:** Potencies of selected thiazolopyrimidines at hA_2A_ AR.

	hA_2A_ IC_50_ (nM) ^a^		hA_2A_ IC_50_ (nM) ^a^
**5**	88 ± 21	**13**	35 ± 8.5
**8**	39±9	**19**	51 ± 12
**9**	7.7 ± 1.3	**18**	14 ± 3.4 (360 ± 115) ^b^
**10**	125 ± 33		

^a^ IC_50_ values of the inhibition of NECA-stimulated adenylyl cyclase activity in CHO cells expressing hA_2A_ AR. ^b^ IC_50_ values of NECA-stimulated activity in CHO cells expressing hA_1_ AR. Data are expressed as means ± standard errors.

**Table 3:** Interaction energies (values in kcal/mol) between compounds bearing the same 2-substituent and a furyl (**8**, **18**, **4**), a 5-methylfuryl (**9**, **19**, **5**), or a phenyl (**6**, **15**, **1**) substituent at the 5-position, and the binding site residues located in the proximity of the 5-position. See text for details.

	**8**	**9**	**6**	**18**	**19**	**15**	**4**	**5**	**1**
pKi A_2A_	9.70	10.15	9.40	10.19	9.59	8.75	8.96	9.10	8.55
R^5^	2-furyl	2-furyl-5-CH_3_	C_6_H_5_	2-furyl	2-furyl-5-CH_3_	C_6_H_5_	2-furyl	2-furyl-5-CH_3_	C_6_H_5_
Val84	−0.0	−0.2	−0.1	−0.1	−0.3	−0.1	−0.0	−0.4	−0.2
Leu85	−0.8	−1.1	−0.5	−0.8	−1.1	−0.5	−0.8	−1.1	−0.6
Thr88	−0.0	−0.2	−0.2	−0.0	−0.2	−0.2	−0.0	−0.3	−0.3
Phe168	−5.0	−4.9	−5.3	−5.7	−5.4	−5.8	−3.9	−4.0	−2.9
Met177	−2.0	−2.3	−0.1	−2.0	−2.3	−0.1	−2.1	−2.3	−0.1
Trp246	−0.6	−1.3	−0.5	−0.6	−1.3	−0.5	−0.6	−1.3	−0.6
Leu249	−2.5	−2.7	−2.1	−2.4	−2.7	−2.0	−2.5	−2.8	−2.0
His250	0.1	−107	−0.6	0.1	−0.3	−0.7	0.1	−0.3	−0.7
Asn253	−7.0	−6.9	−4.9	−6.9	−6.9	−4.9	−7.3	−7.1	−4.4
Ile274	0.6	0.3	−0.2	0.5	0.3	−0.2	0.6	0.3	0.1
tot	−17.2	−21.0	−14.5	−17.9	−20.2	−15.0	−16.5	−19.3	−11.7

**Table 4:** Antidepressant-like effect of **18** in the mouse forced swimming test.

Treatment	Immobility Time (s) ^a^
vehicle	193.7 ± 8.9
**18** 10 mg kg^−1^ p.o.	184.7 ± 5.6
**18** 30 mg kg^−1^ p.o.	143.5 ± 9.1 **
amitriptyline 15 mg kg^−1^ s.c.	149.4 ± 4.6 **

**18** was administered per os (p.o.), amitriptyline was administered subcutaneously (s.c.). ^a^ Each value is the mean ± SEM of 10 mice per group, performed in two different experimental sets. ** *p* < 0.01 vs. vehicle treated animals.

**Table 5:** Antidepressant-like effect of **18** in the mouse tail suspension test.

Treatment	Immobility Time (s) ^a^
vehicle	99.0 ± 6.8
**18** 10 mg kg^−1^ p.o.	88.9 ± 10.16
**18** 30 mg kg^−1^ p.o.	56.5 ± 6.1 **
amitriptyline 15 mg kg^−1^ s.c.	61.6 ± 8.4 **

**18** was administered per os (p.o.), amitriptyline was administered subcutaneously (s.c.). ^a^ Each value is the mean ± SEM of 10 mice per group, performed in two different experimental sets. ** *p* < 0.01 vs. vehicle treated animals.

**Table 6:** Antianhedonic like effect of **18** in the mouse sucrose preference test.

Treatment	Sucrose Preference (%), 6 h ^a^	Sucrose Preference (%), 24 h ^a^
vehicle	78.1 ± 2.0	75.3 ± 1.8
Vehicle + LPS	42.3 ± 3.1 ^^	38.0 ± 5.6 ^^
LPS + 18 10 mg kg^−1^ p.o.	48.9 ± 6.7	39.7 ± 3.7
LPS + 18 30 mg kg^−1^ p.o.	67.3 ± 1.6 **	64.9 ± 2.6 **
LPS + amitriptyline 15 mg kg^−1^ s.c.	69.1 ± 4.8 **	70.3 ± 6.4 **

**18** was administered per os (p.o.), amitriptyline was administered subcutaneously (s.c.). ^a^ Each value is the mean ± SEM of 10 mice per group, performed in two different experimental sets. ^^ *p*< 0.0 vs. vehicle + LPs; ** *p* < 0.01 vs. vehicle treated animals.

## Data Availability

Data is contained within the article.

## Supplementary Materials

The following are available online at https://www.mdpi.com/article/10.3390/ph14070657/s1, ^1^H NMR and ^13^C APT NMR spectra of some selected derivatives (**8**, **10**, **11**, **17**, **18**).
