# Supplementary material for: Design and Synthesis of Novel Thiazolo[5,4-d]pyrimidine Derivatives with High Affinity for Both the Adenosine A1 and A2A Receptors, and Efficacy in Animal Models of Depression
Source: Pharmaceuticals (Basel). 2021 Jul 9;14(7):657. doi: 10.3390/ph14070657 (PMC8308585; doi:10.3390/ph14070657)
Supplement: Supplementary file 1 [file pharmaceuticals-14-00657-s001.zip › pharmaceuticals-1282633-supplementary.pdf]

Supplementary Material

Design and synthesis of novel thiazolo[5,4-d]pyrimidine derivatives with high affinity for both the adenosine A_1 and A_{2A} receptors, and efficacy in animal models of depression.

Flavia Varano [1,*], Daniela Catarzi [1], Erica Vigiani [1], Diego Dal Ben [2], Michela Buccioni [2], Gabriella Marucci [2], Lorenzo Di Cesare Mannelli [3], Elena Lucarini [3], Carla Ghelardini [3], Rosaria Volpini [2] and Vittoria Colotta [1]

1 Dipartimento di Neuroscienze, Psicologia, Area del Farmaco e Salute del Bambino, Sezione di Farmaceutica e Nutraceutica, Universita'degli Studi di Firenze, Via Ugo Schiff 6, 50019 Sesto Fiorentino (FI), Italy; daniela.catarzi@unifi.it (D.C.); erica.vigiani@unifi.it (E.V.);vittoria.colotta@unifi.it (V.C.)
2 Scuola di Scienze del Farmaco e dei Prodotti della Salute, Università degli Studi di Camerino, Via S. Agostino 1, 62032 Camerino, MC, Italy; diego.dalben@unicam.it (D.D.B.); michela.buccioni@unicam.it (M.B.); gabriella.marucci@unicam.it (G.M.); rosaria.volpini@unicam.it (R.V.)
3 Dipartimento di Neuroscienze, Psicologia, Area del Farmaco e Salute del Bambino, Sezione di Farmacologia, Università degli Studi di Firenze, Viale Pieraccini 5, 50139 Firenze, Italy; lorenzo.mannelli@unifi.it (L.D.C.M.); elena.lucarini@unifi.it (E.L.) carla.gherardini@unifi.it (C.G.)
* Correspondence: flavia.varano@unifi.it

Contents:

^{1}H NMR and ^{13}C APT NMR spectra of some selected derivatives (**8**, **10**, **11**, **17**, **18**)

The ^{13}C APT (Attached Proton Test) experiment distinguishes between C-H multiplicities. CH and CH_3vectors have opposite phases compared to C and CH_2. Therefore, the phase of CH and CH_3 peaks is 180° different from C (including the solvent DMSO) and CH_2 peaks.

Compound **8**

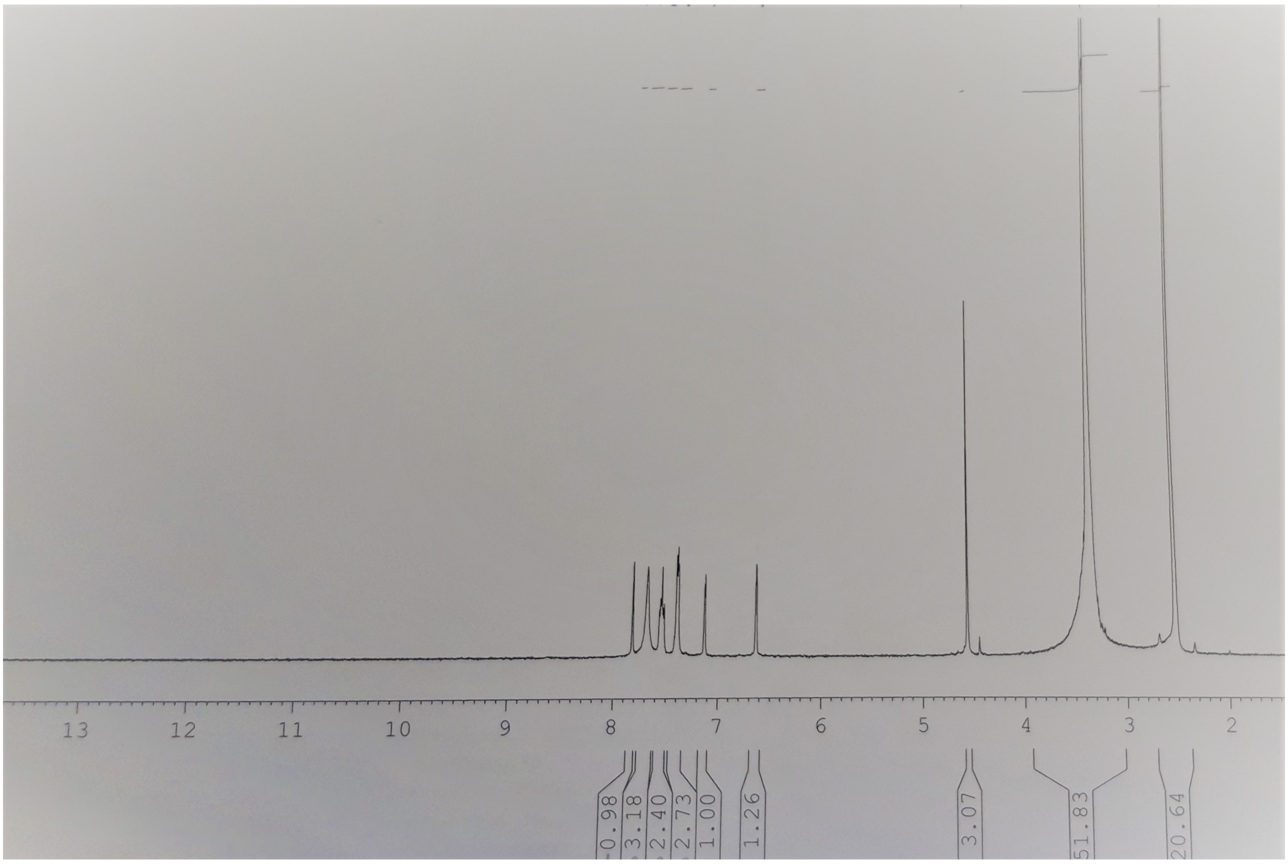

13
12
11
10
9
8
7
6
5
4
3
2
0.98
3.18
2.40
2.73
1.00
1.26
3.07
51.83
20.64

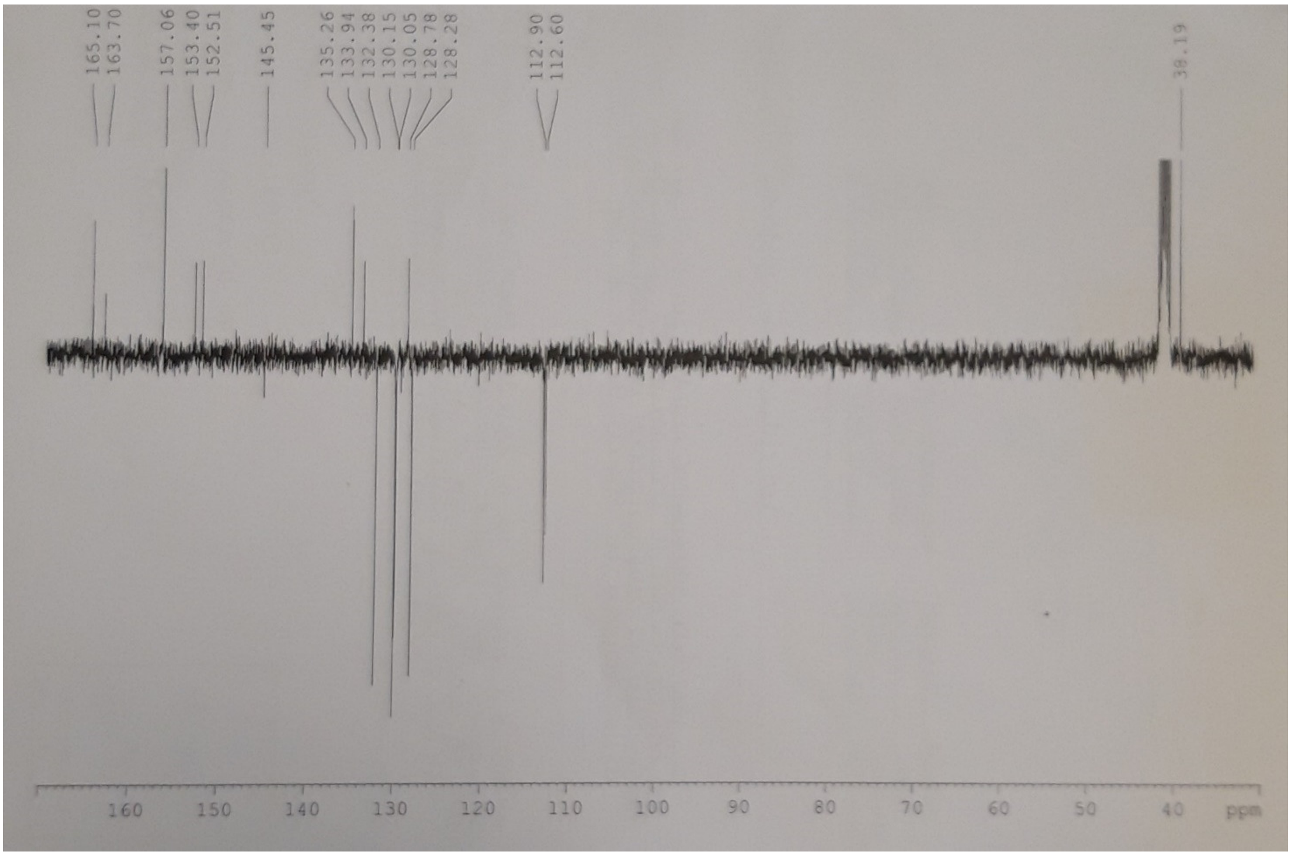

165.10
163.70
157.06
153.40
152.51
145.45
135.26
133.94
132.38
130.15
130.05
128.78
128.28
112.90
112.60
38.19
160
150
140
130
120
110
100
90
80
70
60
50
40
ppm

compound **10**

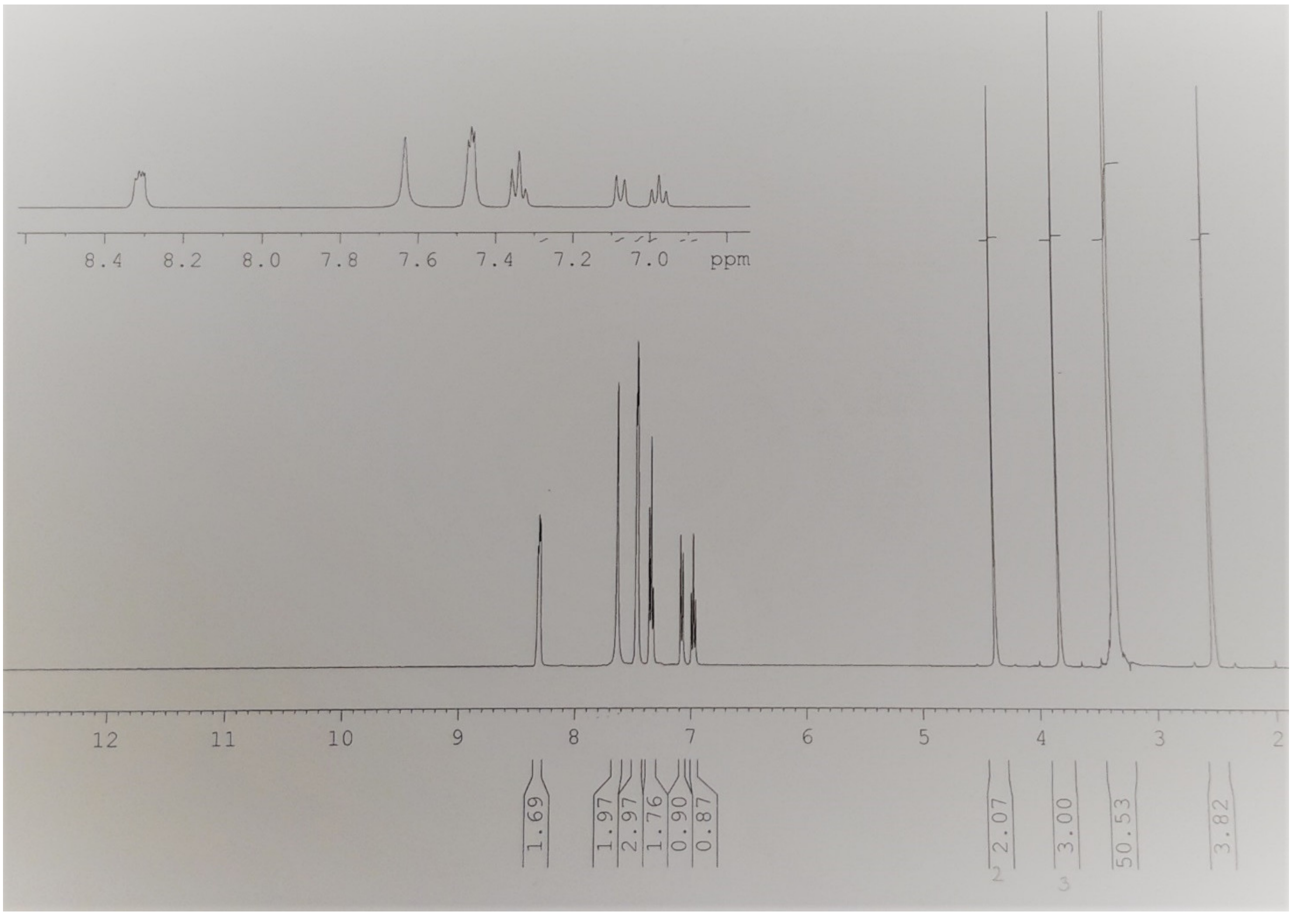

8.4
8.2
8.0
7.8
7.6
7.4
7.2
7.0
ppm
12
11
10
9
8
7
6
5
4
3
2
1.69
1.97
2.97
1.76
0.90
0.87
2.07
3.00
50.53
3.82

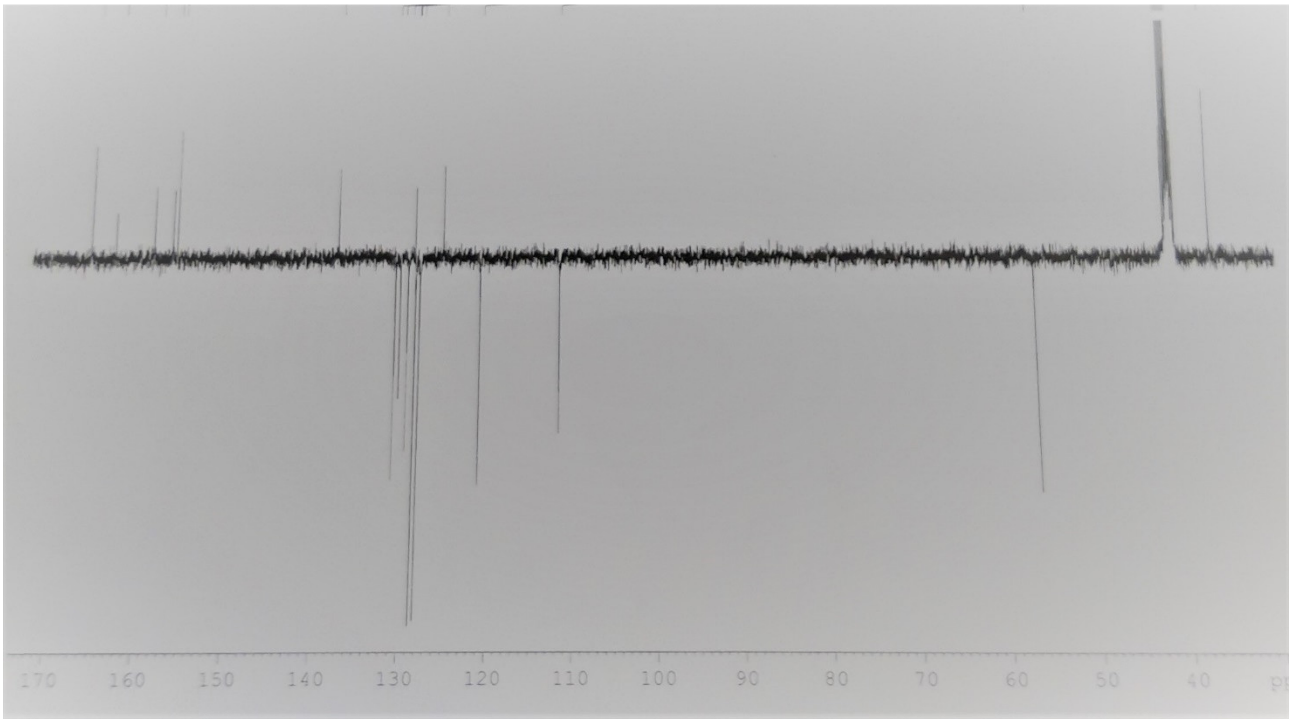

170
160
150
140
130
120
110
100
90
80
70
60
50
40

Compound **11**

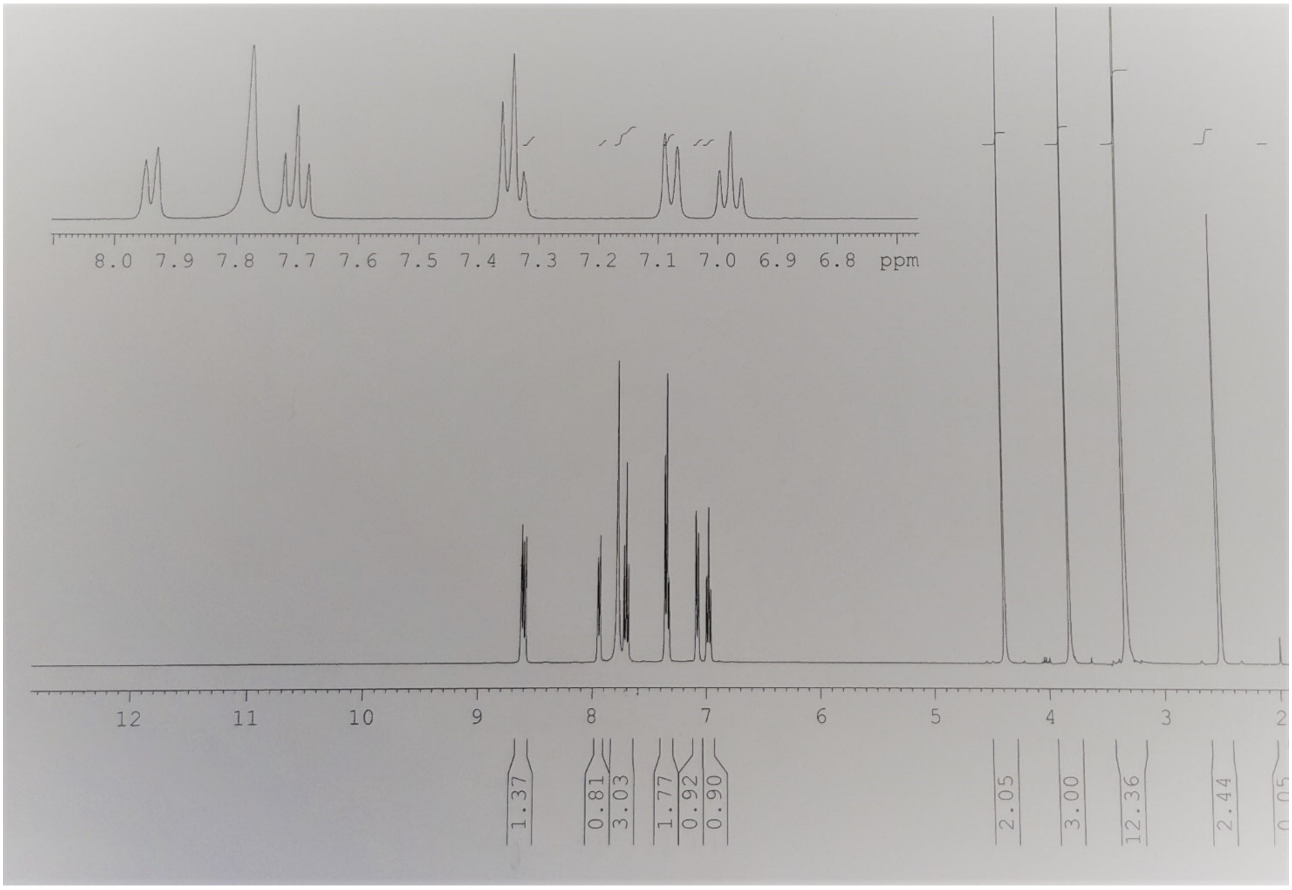

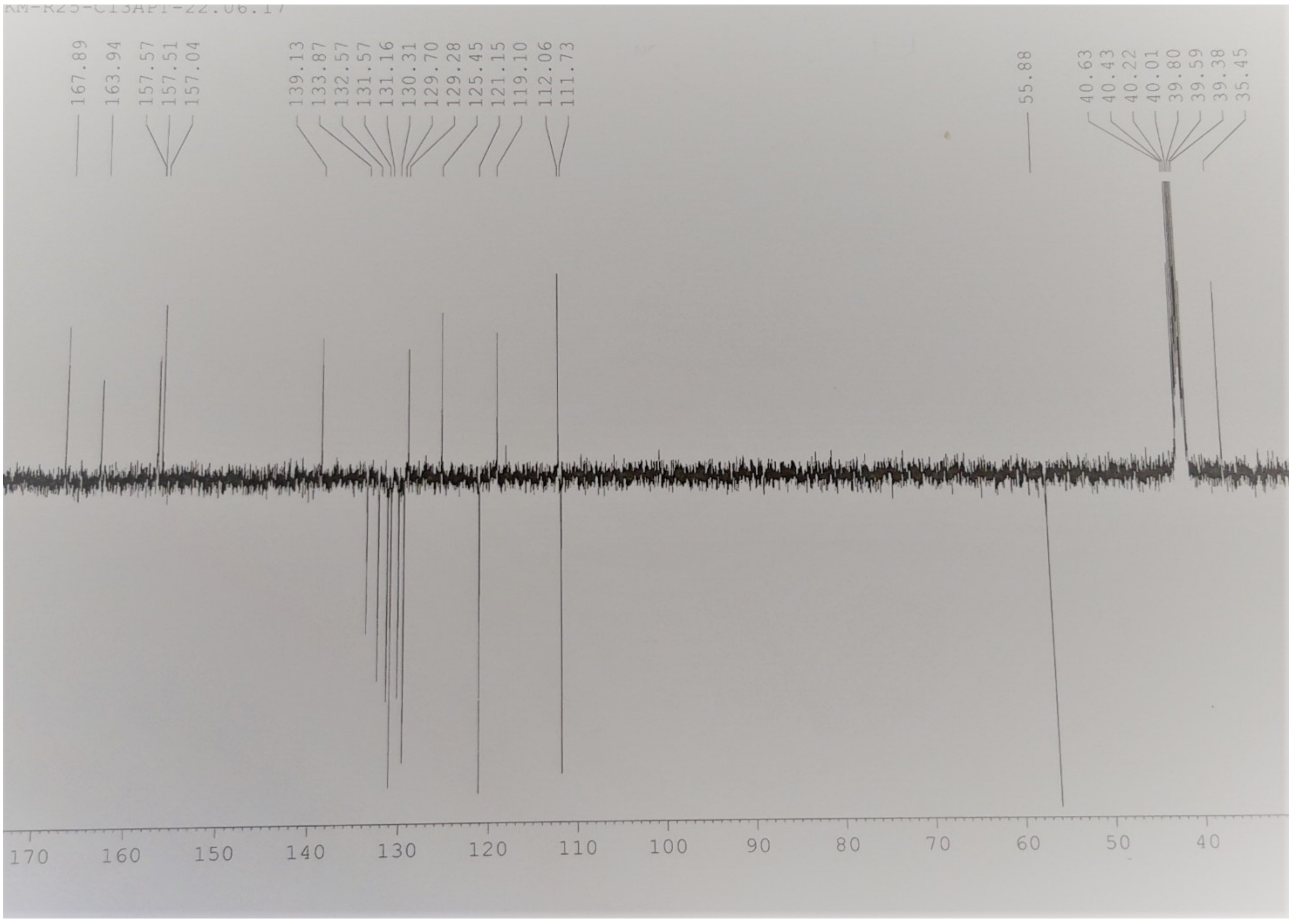

Compound **17**

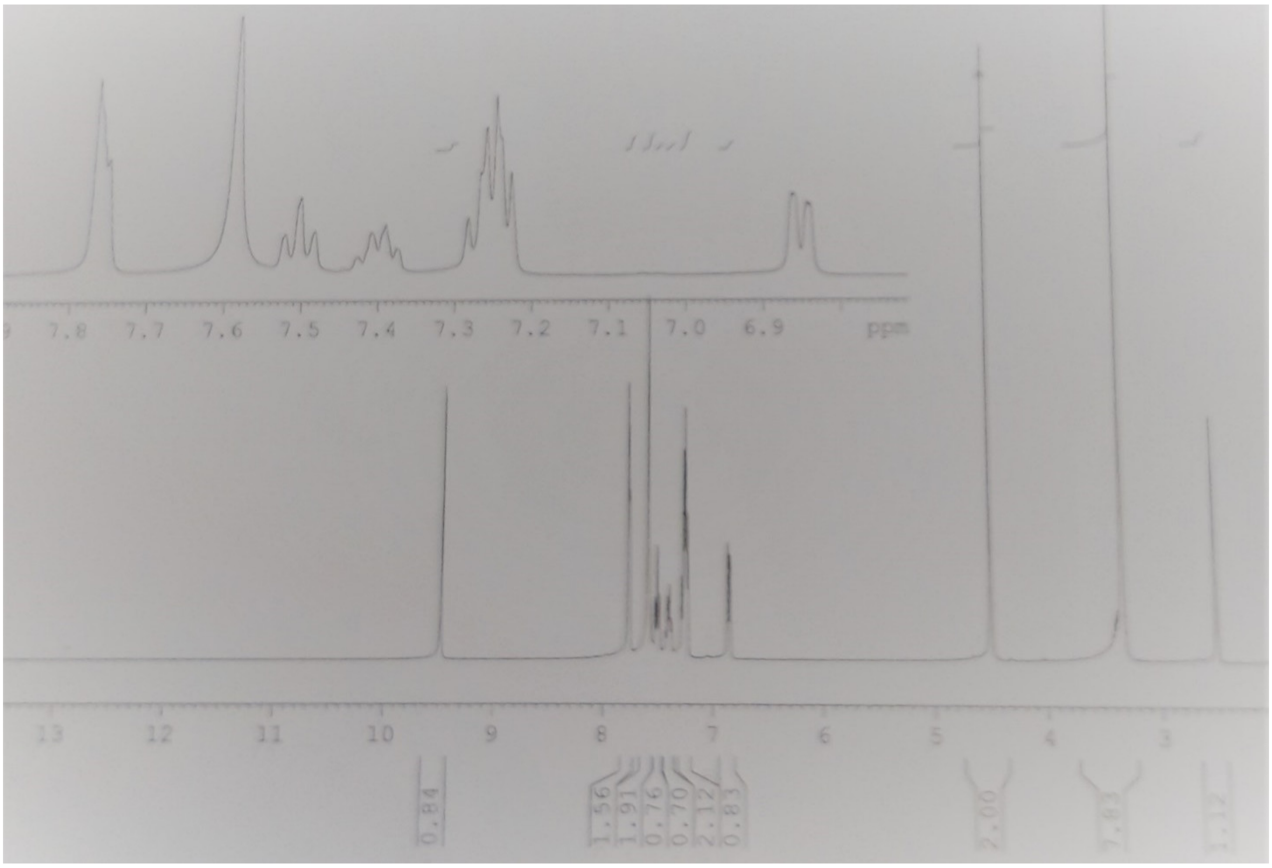

7.8
7.7
7.6
7.5
7.4
7.3
7.2
7.1
7.0
6.9
ppm
13
12
11
10
9
8
7
6
5
4
3
0.84
1.56
1.91
0.76
0.70
2.12
0.83
2.00

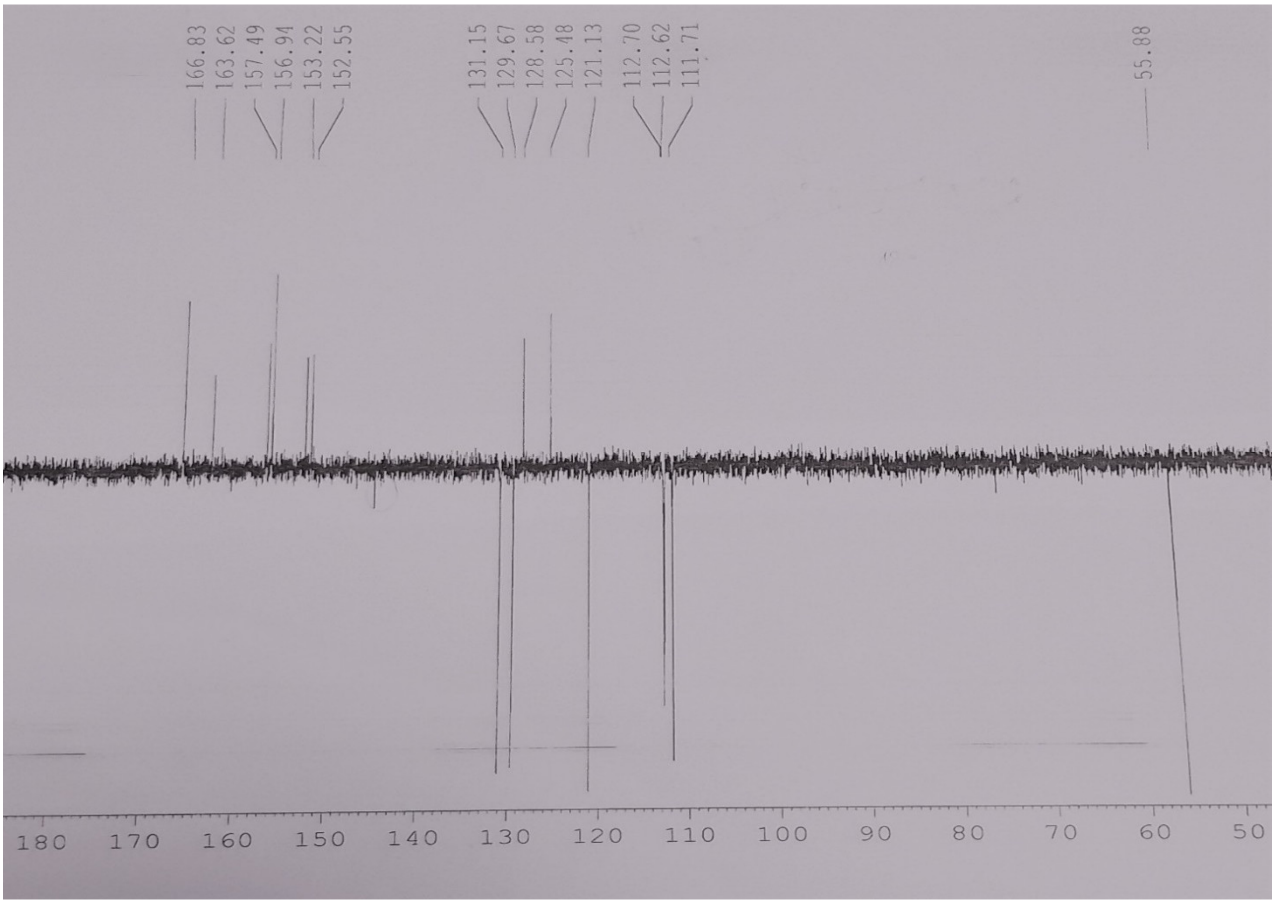

166.83
163.62
157.49
156.94
153.22
152.55
131.15
129.67
128.58
125.48
121.13
112.70
112.62
111.71
55.88
180
170
160
150
140
130
120
110
100
90
80
70
60
50

Compound **18**

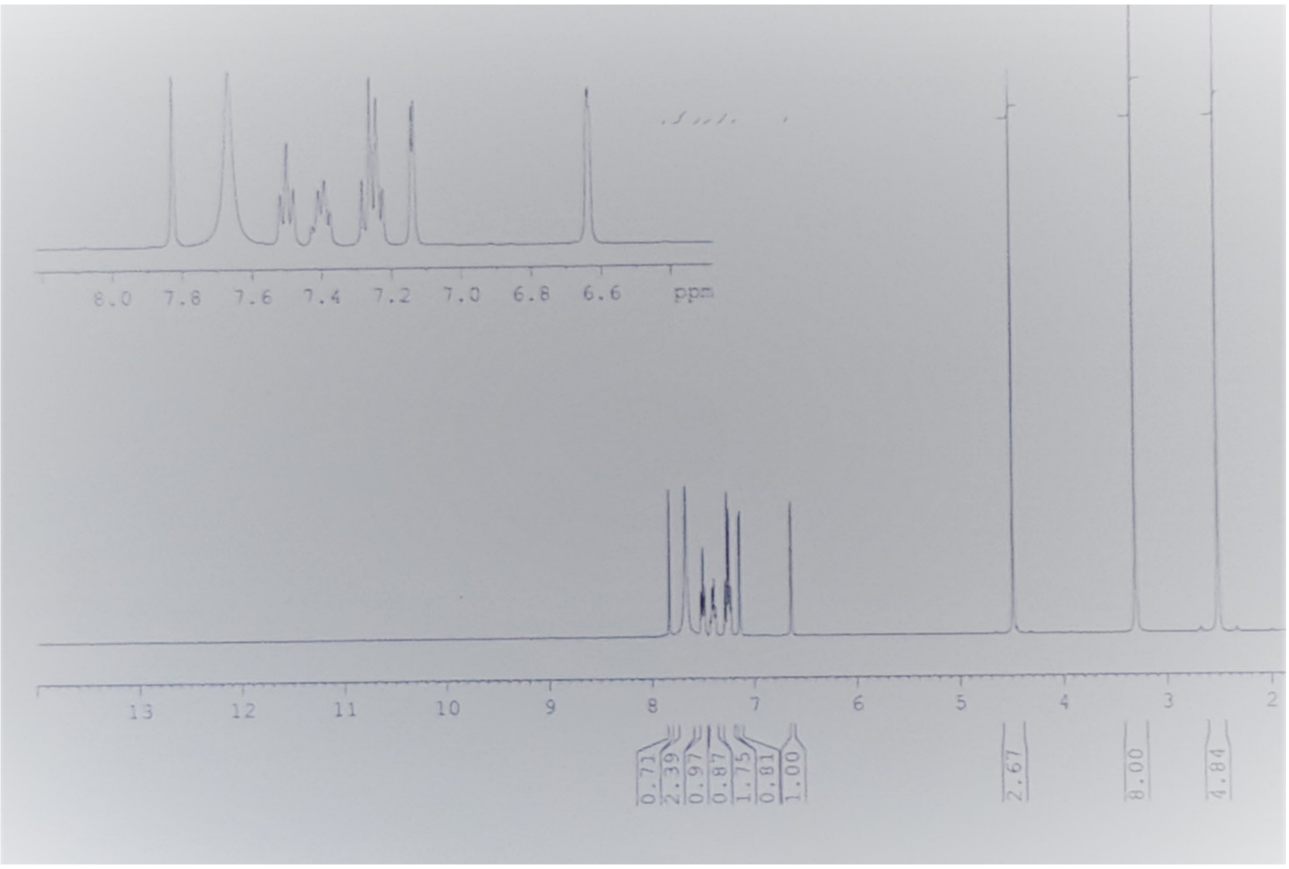

8.0 7.8 7.6 7.4 7.2 7.0 6.8 6.6 ppm
13 12 11 10 9 8 7 6 5 4 3 2
0.71 2.39 0.97 0.87 1.75 0.81 1.00
2.67
8.00
4.84

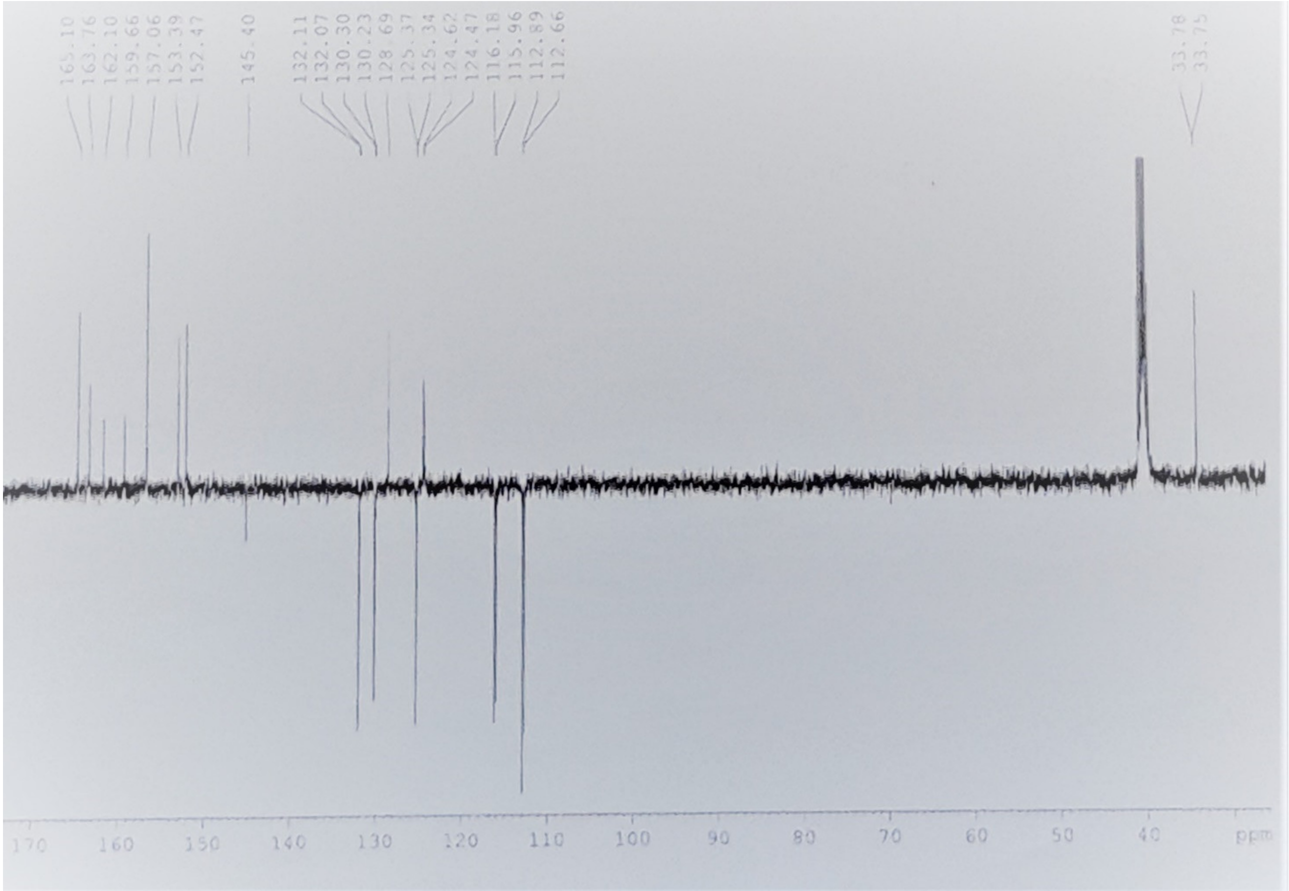

165.10 163.76 162.10 159.66 157.06 153.39 152.47
145.40
132.11 132.07 130.30 130.23 128.69 125.37 125.34 124.62 124.47 116.18 115.96 112.89 112.66
33.78 33.75
170 160 150 140 130 120 110 100 90 80 70 60 50 40 ppm
